# Development of Genic and Genomic SSR Markers of Robusta Coffee (*Coffea canephora* Pierre Ex A. Froehner)

**DOI:** 10.1371/journal.pone.0113661

**Published:** 2014-12-02

**Authors:** Prasad S. Hendre, Ramesh K. Aggarwal

**Affiliations:** Centre for Cellular and Molecular Biology (CSIR-CCMB), Hyderabad, Telangana, India; Mahatma Phule Agricultural University, India

## Abstract

Coffee breeding and improvement efforts can be greatly facilitated by availability of a large repository of simple sequence repeats (SSRs) based microsatellite markers, which provides efficiency and high-resolution in genetic analyses. This study was aimed to improve SSR availability in coffee by developing new genic−/genomic-SSR markers using *in-silico* bioinformatics and streptavidin-biotin based enrichment approach, respectively. The expressed sequence tag (EST) based genic microsatellite markers (EST-SSRs) were developed using the publicly available dataset of 13,175 unigene ESTs, which showed a distribution of 1 SSR/3.4 kb of coffee transcriptome. Genomic SSRs, on the other hand, were developed from an SSR-enriched small-insert partial genomic library of robusta coffee. In total, 69 new SSRs (44 EST-SSRs and 25 genomic SSRs) were developed and validated as suitable genetic markers. Diversity analysis of selected coffee genotypes revealed these to be highly informative in terms of allelic diversity and PIC values, and eighteen of these markers (∼27%) could be mapped on a robusta linkage map. Notably, the markers described here also revealed a very high cross-species transferability. In addition to the validated markers, we have also designed primer pairs for 270 putative EST-SSRs, which are expected to provide another ca. 200 useful genetic markers considering the high success rate (88%) of marker conversion of similar pairs tested/validated in this study.

## Introduction

Coffee tree belongs to the genus *Coffea*, comprising two main cultivated species *C. arabica* L. (2n = 4x = 44) and *C. canephora* Pierre ex A. Froehner (diploid, 2n = 2x = 22), yielding arabica and robusta type of coffees, respectively. Arabica coffee is known for excellent cup quality but suffers from a narrow genetic base due to its domestication history and susceptibility to diseases and pests. In contrast, robusta coffee though poor in quality has better adaptability to various stresses. To keep pace with the environment and also of the sensibilities of market, there is a continuous need for genetic improvement of coffee, which unfortunately is severely constrained owing to inherently slow pace of tree breeding using conventional methods, and variety of other reasons [1], [2]. The situation demands development of new, easy, practical technologies that can provide acceleration, reliability and directionality to the breeding efforts, as well as characterization of cultivated/secondary gene pool for proper utilization of the available germplasm in coffee genetic improvement programs. In this context, DNA polymorphism based genetic markers becomes important that have proven to be of immense value in characterization and genetic improvement of plant germplasm resources.

Among different types of DNA markers, microsatellites or SSR markers are the most ideal for studying genetic diversity, population structure, phylogenetic relationships, construction of frame-work linkage maps, QTL interval mapping, marker-assisted selection (MAS), etc., thereby aiding in genetic improvement of crop plants [1]. In the last few years a number of efforts have lead to development of a few hundred SSR markers in coffee [2]–[12], but these are insufficient to realize the full potential of markers for mapping/linkage studies in coffee, more so in arabicas which have an extremely narrow genetic base. Moreover, most of the described markers are poorly validated, especially for their utility in cultivated genepool comprising arabicas and robusta coffee. The situation thus calls for newer efforts to generate additional validated markers for them being of any gainful utility in marker-based genetic studies/coffee breeding.

With advancements in genomic studies, there has been an huge burst in the EST sequences in the public domain that provide an easy and economic/cost-effective opportunity to identify and develop EST based SSR markers, which have the additional advantage of assessing the functionally effective genetic diversity [13], [14], and also have very high cross-species transferability [8], [11]. In this study, we have used the coffee EST database containing 13,175 unigene [15] to identify SSRs in the expressed part of coffee genome, and use the same to develop novel coffee-specific EST-SSRs for use as efficient genetic markers. Thus we describe here 44 new validated genic-SSRs, and another set of 270 putative similar markers that need further validation. In addition, we also describe 25 new genomic SSR markers that were developed using an affinity capture approach based SSR-enriched partial, small-fragment genomic library.

## Materials and Methods

### Plant material and DNA extraction

The plant material used for the validation of SSR markers comprised a set of 16 elite coffee genotypes belonging to *C. arabica* (tetraploid arabicas) and *C. canephora* (diploid robustas) and 14 related wild species belonging to *Coffea* and *Psilanthus* [2] that were available in the Coffee Germplasm Bank maintained at Central Coffee Research Institute, Balehonnur, Karnataka, India. The fresh leaf samples collectedfrom each genotype were used for DNA isolation as described by Aggarwal et al. [16]. The DNA isolated from robusta variety CxR was used for constructing SSR enriched small-insert genomic library.

### Microsatellite screening of coffee transcriptome, identification of SSRs and marker development

An EST database of robusta coffee comprising 13,175 unigene ESTs [15] was downloaded from ftp site (ftp://ftp.sgn.cornell.edu/coffee/) maintained by Sol Genomics Network (SGN, http://www.sgn.cornell.edu/coffee.pl). The database was used for: (i**)** identification and localization of SSRs using microsatellite search module MISA (MIcroSAtellite, http://www.pgrc.ipk-gatersleben.de/misa), and the criteria being- a minimum repeat core of 12 bp, considering the base complementarities and a minimum distance of 50 bp between two SSRs; (ii) selecting the ‘usable/candidate SSRs’ for marker development, being those that carried a minimum of 18-bp long repeat core (nine repeat units of DNRs, six of TNRs, five of TtNRs, four of PNRs, or three of HNRs) (iii) designing of primer pairs for the selected usable SSR sequences using PRIMER 3 tool embedded in MISA and/or GENETOOL Lite version 1.0 (http://www.biotools.com/downloads/brochures/GeneTool2.pdf); and (iv) standardizing PCR conditions followed by validation of working primer pairs for genetic studies as described earlier [2].

### Construction of an SSR-enriched small-insert genomic library/development of genomic SSRs

A partial genomic DNA library enriched for microsatellite repeats was constructed using the methods described earlier [17]. Briefly, the method involved: one-step restriction digestion of genomic DNA with *Hae* III enzyme (NEB) and ligation of resulting fragments with ds *Mlu*-I adaptor (*Mlu*-F: CTC TTG CTT ACG CGT GGA CTA and *Mlu*-R: pTAG TCC ACG CGT AAG CAA GAG CAC) [18]; amplification of the restricted-ligated DNA pool using *Mlu*-F primer; SSR enrichment of the amplified DNA pool using liquid phase hybridisation (in 6X SSC) with streptavidin coated paramagnetic beads (Dynal) attached with biotinylated equimolar pool of four oligos (CA)_15_, (GA)_15_, (GAA)_15_ and (CAA)_15_. This was followed by amplification of the hybridized/trapped genomic DNA fragments by PCR and construction of partial genomic library in TA vector (Invitrogen) as per the manufacturer’s instructions. A number of positive (white) recombinant clones were randomly picked up from the library, amplified and sequenced for both the strands using M13 universal primers on ABI 3730 DNA Analyzer (Applied Biosystems, USA). The sequences were aligned and edited using Autoassembler (Applied Biosystems, USA). The SSR-positive sequences were identified and used for development of new genomic SSRs as described earlier [2].

The amplified PCR products generated using all the new SSRs were resolved using capillary-based ABI 3730 DNA Analyzer and were precisely sized for major, comparable and conspicuous peaks using GeneMapper 3.7 (Applied Biosystems), using default parameters.

### Statistical, genetic and diversity analysis

The data for EST-SSRs and genomic SSRs were analyzed separately for various genetic parameters, *viz*., mean, standard deviation, expected heterozygosity (*H_e_*), Hardy-Weinberg equilibrium (HWE) and linkage disequilibrium, (LD), using Arlequin ver 3.1 [19], polymorphism information content (PIC) and private alleles (Pas) using Convert ver. 1.3.1 [20]. Cross-taxa transferability (T*_mark_*) was calculated over 15 species (except *C. canephora*) as proportion of primers showing successful amplification *vis-à-vis* all the tested primers whereas primer conservance (C*_taxa_*) was calculated as proportion of the species displaying successful amplification *vis-à-vis* all the tested markers.

Genetic diversity analysis to infer generic relatedness/affinities was performed over informative P*m*s (polymorphic markers) for cultivated genotypes/related species using MicroSatellite Analyzer [21] with Nei’s genetic distance [22]. The genetic distance matrices were used to construct Neighbour Joining (NJ) consensus tree using Phylip ver 3.6 [23], which was viewed using Treeview ver 1.6.6 [24].

We also attempted mapping of the new markers on a robusta linkage map using JoinMap ver 4.0 [25] as described earlier [2], [26] using group LOD score of 5.0.

## Results

In this study, we undertook *in-silico* analysis of a robusta coffee transcriptome to identify and develope coffee-specific EST-SSR markers. Simultaneously, we also attempted development of genomic-SSRs by constructing a small-insert SSR enriched partial genomic DNA library. The new markers (44 EST-SSRs and 25 genomic-SSRs) were validated for their utility in genetic studies using panels of elite coffee genotypes, and related taxa of coffee for cross-species transferability. The details of these new markers *viz*., locus designation, primer sequences, repeat motifs, amplification temperature, amplicon size, and SGN ID or Genbank accession numbers are given in Tables 1 and 2. Details of additional primer pairs for 270 putative EST-SSRs, which were designed but need to be validated have been provided in Table 3.

**Table 1:** Details of the new EST-SSR markers developed using EST database and validated using diverse coffee genotypes in the present study.

Sl. No.	Marker Id	Primer sequence	Repeat motif	Allele size (bp)*	Source EST ID**	Linkage group
1	**CCESSR01**	F: TGGTAGCACTGTCGGAAGCATAT	(AGC)7	239	119516	–
		R: GACCCATCTAACTTGCTGCATTTT				
2	**CCESSR02**	F: AAGATATGTTTTAGCCCAAGTAGTGAC	(AT)11	168	119534	–
		R: ATTGGTTGGTACTGTTTAGCTGTTCAT				
3	**CCESSR03**	F: CAGCCGTATCAGCACCAGCAT	(TC)10	217	119559	CLG02
		R: TTCCCAACCCGTCAAAGTCCT				
4	**CCESSR04**	F: GTCGACAACAGCGCTCAAGATC	(TCT)6	259	119613	–
		R: CAAAAAAGACTGGAAAGAGGGTATTAG				
5	**CCESSR05**	F: AGGGGCTGGTTATTTTTTGGG	(GCA)6	141	119723	–
		R: GGGGGTAAATACGGGAAAGCAGA				
6	**CCESSR06**	F: AACCCTCCCTCCTCCACCTTTTC	(AC)23	224	119781	CLG09
		R: GGAGGTGGTGGTGGTGTAAAAAAAG				
7	**CCESSR07**	F: CCCCCTTCCCATTTTTTCCCCT	(AGC)6	194	119864	–
		R: CCGGGTGGTAGAGAGATTGCTGCA				
8	**CCESSR08**	F: AAACCACAACATCCCCCAAGAGT	(GCA)8	149	120008	–
		R: GGCAGTAGAATTGGAGCGGTGAAA				
9	**CCESSR09**	F: CCCCCACCCACTTCTCTTTG	(TTC)8	184	120113	–
		R: ACAACAAACGAACGCTCTCTGATAA				
10	**CCESSR10**	F: GCAGAAGAAGCACCAGTAGCAGAAGAAG	(GCT)7	207	120120	–
		R: TGCCTTCTACTCTTCACTCTTCTCCACT				
11	**CCESSR11**	F: AGGGAAAAAGAAGAGTGAGAAGAATATT	(GAA)6	140	120217	–
		R: GGGACACTCACATATACTGCAAACTTAG				
12	**CCESSR12**	F: CCGCCATCCCTTTTGCCTTTC	(CCA)6	200	120227	CLG02
		R: ACGGCAGCAGAAGTGGAGGTGTT				
13	**CCESSR13**	F: GCGGGGTAGTTTTGGGAATATGG	(TTA)6-tt-(TTC)6	124	120252	CLG02
		R: TTTGGGGTCCTTTTTCTTTCACACAT				
14	**CCESSR14**	F: CTTGCCCCCTTCCCTCCCACTC	(CT)10	232	120329	–
		R: TTCGGCTCCTTGTGTTTGGGTA				
15	**CCESSR16**	F: AGAGCAATGAGAAACAAACGAAACT	(CAT)6	173	120439	–
		R: AGGTGCCCAACTATCCCAGAAT				
16	**CCESSR17**	F: CTCCACACCAACAAAATCCCACTT	(CAG)6	169	120475	–
		R: CCCACATCCTGAGTCTGCTGCTAA				
17	**CCESSR18**	F: GGGGAGGATGCTTATGATATGAGG	(CAC)6	152	120498	–
		R: TCCGGTTCACCTGCTTTTCCTT				
18	**CCESSR19**	F: CATCGTATCTCGCCCTCTCTCTTTC	(CA)13	219	120514	–
		R: CCACAACAAGTACAACCAACCGAAAC				
19	**CCESSR20**	F: TTCTGGCCGATTGATTGTGAT	(AAG)7	143	120538	–
		R: GCGACAAGGCTGACAAACTACTAC				
20	**CCESSR21**	F: CGAGCTAGTGCAGACAGATTGAGAT	(AG)17	164	120568	CLG13
		R: GTCCTTGGCGAAATCCCTCAG				
21	**CCESSR22**	F: CCCTCAATCTCGTCCCCCTCT	(TC)9	153	120823	–
		R: CCCTCCATAAATCTTCTTCACGTACTC				
22	**CCESSR23**	F: GGCCTCTCTTTAATTTTCTTGTCTTTTTTC	(TTC)8	160	120860	–
		R: ATGGAGGGTAGGGTTTCGAGAGTGA				
23	**CCESSR26**	F: AACCGGCCTTCTTGTATGATTCTCTA	(CAT)8	206	121392	CLG07
		R: TTGGCTAACCCTCACTCTCTCCCTACTA				
24	**CCESSR27**	F: GCCCACTCCATTCGTACTTGTTTCC	(CT)12	120	121464	–
		R: GCGGTGCTGCTCAATGCTCAT				
25	**CCESSR28**	F: AAAATGAGTGACGATGGGAAAGACA	(CCA)7	193	121482	–
		R: GAGGGAAGCCGATCACTGGTTG				
26	**CCESSR29**	F: GGCGCTAGAGTTGGTTGTTTGC	(CTTCT)5	95	121548	–
		R: CAGGCATTGGAACCAGCGAAC				
27	**CCESSR31**	F: AGAAGAGTACTGAAGGCCTGGAAGA	(GTG)6	220	121671	–
		R: AGCATCTGCAGCCTCCATAGC				
28	**CCESSR32**	F: CTTGGCGTTTAGCGTTCTCACATT	(TA)9	163	121811	–
		R: GCTCAACCAAACCAATACATACCTCTT				
29	**CCESSR33**	F: GCCCGCATGGACGACTTGGA	(AGG)7	227	121876	CLG13
		R: CGCTTGACGTATCCTTTGGCCTCT				
30	**CCESSR34**	F: GCATTGCTCCCCCCACTTCA	(CTC)6	168	121905	–
		R: GAGCATGGGGACGAGGAGGA				
31	**CCESSR35**	F: CTGCTAATGCTGCTGAAAAAGAGATACC	(AG)10	107	121994	CLG13
		R: GGCTGTGAATTCTTGTGACTTGTGACT				
32	**CCESSR36**	F: AGCCTTCTGCAATTCCCTCGTACA	(AG)9	100	122089	–
		R: GGCGTCGTAGAGGGCATTCAGA				
33	**CCESSR38**	F: GCCCGAGGGTTAGATTGATCA	(AG)12	162	122114	–
		R: CTTGTCTTCTGTTTGATTTTGTGTTCTA				
34	**CCESSR39**	F: GCGACCGGACGACCAAAAATAAT	(GAA)7	131	122147	–
		R: CGCCGTCGTCAGAGTCATAATAATCA				
35	**CCESSR40**	F: CGTGGGGGTTTGTTTTTCTCG	(AGG)7	205	122194	–
		R: GTCCCCCCTCAGCCGTTTTTG				
36	**CCESSR41**	F: GGGCTGCAGGCTTGTCACCAC	(GA)10	205	122322	–
		R: AATCGGTTTAGTTTTTTGTTTCCTCAC				
37	**CCESSR42**	F: CGGGCGGAAACGGTCAGATC	(GCA)7	117	122295	–
		R: TGCCGTTGTTGTTGTCCAGGTG				
38	**CCESSR43**	F: CCCAGCAAGAACTCAACCCCATCA	(TTC)8	172	122653	CLG02
		R: TGGCCTAATGAAGATGACGTTGCTGATG				
39	**CCESSR44**	F: AGGAATAATGGAGGAGACGTTGTTG	(CTT)7	236	122680	CLG02
		R: GCACAAATCCCAGTACTTCCTCATAGA				
40	**CCESSR45**	F: AAATGGCCGAGATAGAGAAGGAGAAG	(AGG)7	135	122764	–
		R: CCCACTCCTCCGCGGTACTGATC				
41	**CCESSR47**	F: GCAGCAACAATCACTTCCACAGC	(GCA)6	198	122922	–
		R: TGCTGTTGTAACTGCGGGATTTG				
42	**CCESSR48**	F: GCAACCTTATCTAGATTCAACTTCAACTT	(AAATCA)5	188	122975	–
		R: CGGGAAGAAATGGCAGCCTATAC				
43	**CCESSR49**	F: GCGGCCATCCTTGTCTTCG	(ATC)8	186	122978	CLG02
		R: TAGCCGCTGACGTAATCTTCCTT				
44	**CCESSR50**	F: GGGATGATGTGGATTCTATGGTCTACTA	(CAG)7	108	123181	CLG10
		R: ATGCCATTTTAACACTTCCTCCTCA				

CCESSR: **C**CMB **C**offee **E**ST **SSR** marker;

F: forward primer;

R: reverse primer;

–: Unmapped;

CLG: Combined Linkage Group [1];

*: Predicted amplicon size based on source EST sequence;

**: Source EST ID as per the downloaded SGN database (ftp://ftp.sgn.cornell.edu/coffee/).

**Table 2:** Details of the new genomic SSR markers developed using streptavidin-avidin affinity capture SSR-enriched library in the present study.

Sl. No.	Primer Id	Primer sequence	Repeat motif	T_a_ (°C)	Allele size (bp)*	GeneBank acc. No.	Linkage group
1	**CCRM02**	F: AATGGTGGCAGTCCTGAAAGATC	(GA)12	57	268	KM874369	–
		R: AACATCAACTTTCCTGGTCTTC					
2	**CCRM06**	F: TTCTTATCACCTTGGGCTACCTTTCTTC	(AG)8	57	146	KM874370	–
		R: AAGCGGTTTAGTTTTTTGTTTCCTCAC					
3	**CCRM07**	F: TAAAGGATGGTATATGTGGCTGGAGTA	(AT)8	57	126	KM874371	CLG01
		R: CCACAGCCTCGGCATTTACTATATAT					
4	**CCRM10**	F: AAAAAGACAAGATTCAACCTGCAGTAGT	(GT)9	57	104	KM874372	–
		R: TTCCCACCCCCCAAAAAAAA					
5	**CCRM14**	F: ATTTGATTTCTTCTTTCTCTGTTGTC	(CT)22	55	130	KM874373	–
		R: ACAAAAGCCCTGAAAATAATAGATCTA					
6	**CCRM15**	F: CGAAATTGACGAAGCTCTTGTT	(CTT)6	57	243	KM874374	–
		R: TTGCTAGTTTCGAAATCGTGTAAGGAC					
7	**CCRM16**	F: TCCTATAGCAGAAACACAAAATGACACAG	(TC)26	55	223	KM874375	–
		R: GGTTTTTGGGTTCTTTTTAGCATATACA					
8	**CCRM17**	F: TAAGCGTTGGAATTCCTCACTCTATCT	(CT)17	55	228	KM874376	CLG01
		R: ACAGCTAAAGAAACAATGAACCAGT					
9	**CCRM19**	F: GTTTTTTTTTTCTTTTTTCTTTTTGAGCT	(GA)26	57	252	KM874377	CLG06
		R: AAGGCAATGTTGGTCAGCAGTGG					
10	**CCRM21**	F: CACCCCTCCCATCCGTTGAAACAT	(GA)16	57	258	KM874378	CLG06
		R: AATGATGCTCCCAGTGTTTGATGA					
11	**CCRM22**	F: CTTGCAGTTTACTTCCCTTTGGTTG	(GA)29	57	241	KM874379	–
		R: TTTTCTTCTGTATATTGTTGGAGTTCTTC					
12	**CCRM23**	F: CGGCAGTGTGGTCCCCTTTGAAT	(GTT)6	57	141	KM874380	–
		R: AAAAAAAAACTCACACTCTATCAAAACTAAGG					
13	**CCRM24**	F: GAGTGTGAGTTTTTTTTTGTGACCTTAA	(GA)9.(GA)9	57	213	KM874381	–
		R: ACCCCACATTCCTCTCATCCATTC					
14	**CCRM28**	F: GGGGCAACAAGTGGTAGGATATGAAGAC	(CA)10	57	209	KM874382	–
		R: CGCCTTCACTATGGTTTTGCCTTCTAA					
15	**CCRM31**	F: CTTTTATGTCTATCTGTCTCTGCTTTTC	(CA)10	57	114	KM874383	–
		R: CCTGCAGTAGTTTCACCCTTTATCC					
16	**CCRM33**	F: ACAGCCGTTGAACTTATGGGATTACA	(CT)12	57	118	KM874384	–
		R: ACAAAGGGATGGAGAGGATGGAATATAC					
17	**CCRM34**	F: CCCCAGAACGAAAGGCAATCAT	(GA)10	57	165	KM874385	CLG05
		R: TTGGGACTATTTATACTGGGGAAGAA					
18	**CCRM35**	F: GGGGTTAAATCAGGGGAAAAGTGG	(TG)12	57	144	KM874386	–
		R: AAGCGAGGGAGAGAGCAGCAGATC					
19	**CCRM36**	F: CCATGGGGCAAAAGGCAAATTCTAT	(CT)18	57	171	KM874387	–
		R: TCCAGACCGCCGTTCACGAAGTATA					
20	**CCRM37**	F: TGCTTCCCTTCTCATTCTGGTACTTT	(GT)10	57	146	KM874388	–
		R: AATCCATCAACAACTTCAGCATACCA					
21	**CCRM38**	F: TGAGAATTAAAGCAGCAGGGGTATG	(CA)10	57	204	KM874389	CLG05
		R: GCAAAAAAAGGCAAAAGCATTACATC					
22	**CCRM40**	F: ATTCACGCTTTCATTACTTTTCTC	(CT)29	57	176	KM874390	CLG05
		R: TTTGTATTTCCTTTCCATTTCTTTTGTA					
23	**CCRM41**	F: AGCAGAAACACAAAATGACACAGAGCA	(CT)24	57	161	KM874391	–
		R: AATGGTCCAAGGAAAATGAAAAATGTT					
24	**CCRM42**	F: CGGAGAAGAGCAATATACAAGCAAGG	(GA)13	55	143	KM874392	CLG11
		R: GCCACCCCAGAACTTTTGCAA					
25	**CCRM45**	F: CTTCAAGCAAAATTTTCAACAGCACAG	(GA)10	57	187	KM874393	–
		R: GGCCCTTTTTTAGTCTCACCACATT					

CCRM: CCMB CXR Microsatellite marker;

T_a_: annealing temperature;

F: forward primer;

R: reverse primer;

–: Unmapped;

CLG: Combined Linkage Group [1];

*: Expected amplicon size in the robusta variety CxR.

**Table 3:** Details of primer pairs for additional new EST-SSR markers designed in the present study.

Sl No.	Marker ID	Unigene ID*	SSR repeat unit	Forward Primer	Predicted Tm (°C)forward primer	Reverse Primer	Predicted Tm (°C)reverse primer	Predictedamplicon size (bp)
1	CCESSR51	119463	(AGAGCA)5	AGCAGAGATCGAGACAGAGAGA	59	ACATATCTGAAACCCTCGGC	59	477
2	CCESSR52	119606	(CT)9	AATCGGAGGATTTGTGCTTC	59	GCGCCTAAATCACCCATATT	59	390
3	CCESSR53	119736	(GA)11	CCGCGGTCAGTCTTACTACA	59	ACACAAATCAACACCCATCC	58	266
4	CCESSR54	119787	(GGA)6	CGCAATCTTGAATGAGGAGA	59	TGGGAGGTTGATCATCTGAA	59	341
5	CCESSR55	119820	(CT)11	AGGACAGGAGTGTGATCCCT	59	GCCATGTCCTCCTTTCGTAT	59	376
6	CCESSR56	119897	(TC)11	AGGTTTTTGTGTCCCTTTGG	59	CATCGATGAAAAGCAGCAGT	59	230
7	CCESSR57	119919	(AT)15	GAATTTTGTCAGCCAGAGCA	59	GACGGAAAGATTCTGGCTTT	58	184
8	CCESSR58	119987	(TCC)7	AGCTACGCTAGGCAATTGGT	59	GACAACAACAACAGCCAACA	58	296
9	CCESSR59	120008	(TC)12	GGAACAAGACTCTCTTGCCA	58	TCATCACACAAGGAGGCAAT	59	418
10	CCESSR60	120008	(TG)9	ATTGCCTCCTTGTGTGATGA	59	CCGGTCGATCAACAATCTTA	59	198
11	CCESSR61	120045	(TCT)6	GGTGAAGGGTCCTTACCTGA	59	GAGATGTGCTACTGGCTACTGC	59	232
12	CCESSR62	120064	(GATT)5	CTCTTGTTTCCAACCCAACC	59	CGGACACTGTGAGGAGAGAA	59	320
13	CCESSR63	120107	(TCT)6	AGTCCAGTCCAGTCCTGTCC	59	CCGATATGATTTTGGTGCTG	59	432
14	CCESSR64	120121	(GCT)7	CGAAGTTGTGCAGGATGAAC	59	GGAGCTGCTTGCTCTTCTTT	59	241
15	CCESSR65	120179	(AT)10	GTGATGCTCGGTGTATCTGG	59	ACTAGAGGCCGAGAATTGGA	59	203
16	CCESSR66	120206	(AGA)10	GATGAGCTCCAAAACAAGCA	59	AAAACTTCCCAGGCTTCAGA	59	447
17	CCESSR67	120260	(CTCC)5	GTCGTCTGTTCCTCCTCGAC	60	CCACTAATCCCGAGCAAAAT	59	273
18	CCESSR68	120291	(AAG)8	CCACGCGAATAATCATCAAC	59	AAGCACCTTATCCCCAACAG	59	469
19	CCESSR69	120316	(TCA)6	TTGGAAAACCATAGAAGGGC	59	ACCCTCATCAATCTCTTGCC	59	408
20	CCESSR70	120320	(GCCACC)4	TTAAATCAGCCCTCAAGCCT	59	CTTGAAATCTTGCGCACTGT	59	343
21	CCESSR71	120322	(TTC)6	TTAGAAAAGCTGCGAGACGA	59	TTGACCATTTCCCCTTCTTC	59	342
22	CCESSR72	120543	(AT)9	GATTGCTCTCTTTCTTCGGG	59	TCCGCCCCAGTTAGATTTAT	59	382
23	CCESSR73	120545	(AT)9	GTTTTCCGGCTAGCTTGTTC	59	GTGCATGAGGTGAAAATGGA	60	214
24	CCESSR74	120579	(CCT)7	TGTAATATTGCTTCGCTCGG	59	AAGGGATGATGCCTAGTTGG	59	485
25	CCESSR75	120596	(TCC)7	GGCTCAATCGATAAGCAACA	59	CATGAACATATCCTGGAGCG	59	429
26	CCESSR76	120656	(AAC)6	AAGCGAAGATGGAGAGCAAT	59	CCGCCTTTTGTAGAAGAACC	59	316
27	CCESSR77	120656	(TCA)6	TTTTAGGCCAATCCTTCACC	59	GTATGTGAGGGCGTAACGTG	59	454
28	CCESSR78	120720	(GAA)6	GAGCCCTCTTTTTCCTCCTT	59	CCGTAATGATACAAACCCCC	59	326
29	CCESSR79	120764	(CATATA)4–13 bp-(AT)9	AGCAGCACCAGACTAGCTCA	59	GTCCCAGAAACGAAGATGGT	59	399
30	CCESSR80	120821	(ACAGG)4	CAGAATCCCATATTCCCCAC	59	TTGCTGTCTGATTTCCAAGC	59	324
31	CCESSR81	120883	(TCAAT)4	CAGATTCCGGTGTCACAAAG	59	TTTGCTGCAGTGTCACTTGT	58	107
32	CCESSR82	120965	(TCGAAA)4	GACTATGGATGGCTTTGCCT	59	GGCGGATTGGTTGATAGAAT	59	397
33	CCESSR83	121010	(CCTGCA)4	TGAATCAACTCCTGCTCCTG	59	GGTAGTTCCAGCCACCAGAT	59	115
34	CCESSR84	121086	(TC)11	GGATCAAACGTGGCTAAGGT	59	AAGAAAAGGGCTACAACGGA	59	203
35	CCESSR85	121131	(AAG)6	CCCCGGGCTGCAGAAACAAGT	60	AGCTCCGGTAAGCCTCAATA	59	389
36	CCESSR86	121240	(GCA)6	AACCCTCCACTCCATTTCAG	59	ACTATTGTTGCTGCTGCTGG	59	347
37	CCESSR87	121289	(TTC)6	GAAGCAACCCCTCAACTGAT	59	AGCACCCTCTGCTTCAATCT	59	345
38	CCESSR88	121377	(GT)9	CACGCGGATAATACACCG	59	AGTTGCTCCTGCTTCTCCAT	59	191
39	CCESSR89	121392	(GCT)6	GCACTGTTTTGAATGGTTGG	59	TCCGCACTACAAGTACCGTC	59	159
40	CCESSR90	121439	(GATTA)4	TACTTGAGCGATCAGAACCG	59	TAATCCTGCGTGCTATTTGG	59	455
41	CCESSR91	121548	(CTTCT)5	AGGCGCTAGAGTTGGTTGTT	59	CAAAGTAAGCAGCAGGCATT	58	108
42	CCESSR92	121580	(TC)11	CCTACATCCCACGACCTTCT	59	GTGGTAGTGCTTTGGTGGTG	59	361
43	CCESSR93	121610	(AT)9	CTGCCCTATGATGAATGGTG	59	TCATCTCAAGCATCGTCTCC	59	157
44	CCESSR94	121752	(TGCTCC)4	CCTCTCATGCCAGCAACTAA	59	AAAGCACTGGGAACTGAAGC	59	258
45	CCESSR95	121841	(CT)10	TGGAGCAGAGATTGTCAAGG	59	CCAGCTGGAACTTCCCTG	59	349
46	CCESSR96	122004	(AT)19	ACATTCGAAAACTTGGGGAG	59	CAAGGTCTTGGGTTTGTCCT	59	232
47	CCESSR97	122006	(AG)14	CTCGTGCTGTAACCCTCTCA	59	AGTGTGATGGAACGCGAATA	59	439
48	CCESSR98	122012	(AGC)6	GACGCGCAGTCTTTCAAGTA	59	GCTTCACAGTCGTCTTCCAA	59	373
49	CCESSR99	122077	(CGC)6	TCGGACTGCTATAGTGGACG	59	TCCTTCGAGACGTTAGCCTT	59	215
50	CCESSR100	122089	(AG)9	TTCTGCAATTCCCTCGTACA	59	AGGGGTCAGAAGAAGGGATT	59	214
51	CCESSR101	122184	(AT)10	AGGGGTGGATTCAGTAGACG	59	ATGCAATTGAACCGACTCAG	59	466
52	CCESSR102	122195	(AGGCTC)6	AGTCAGCTTCTTGCTGCTCA	59	CCCTCCAGAGTACCCATTGT	59	336
53	CCESSR103	122254	(TCGT)5	GGAATGAGCTAAAAGCCCAG	59	TTACCGTGTAGGTTGGTGGA	59	318
54	CCESSR104	122257	(TTCA)5	GCTCAGTGAGGTTCCAAACA	59	ACCCTGTAGGTTGGTGGAGA	59	310
55	CCESSR105	122324	(AGA)6	AGGCCATAGTGGTGAACACA	59	ACAAAGCACAGAACCAACCA	59	319
56	CCESSR106	122340	(TC)10	AGCTGATTGAGGATGGCTCT	59	CATTGGCTTTCCCCAATACT	59	484
57	CCESSR107	122383	(CTT)8	CCTTCTCCCTCTTCCCTTCT	59	TGAGTGACAGCGTCAACAAA	59	459
58	CCESSR108	122387	(CTT)7	GTGGGAAATCTCAAATGGCT	59	CTCTTTCTGCTGGGTTGTGA	59	143
59	CCESSR109	122514	(AGG)6	GAAAGACCCCAAACCAAGAA	59	GGAGTCATCAACAATGGCAC	59	340
60	CCESSR110	122533	(TA)10	GTGCCGTTGTTTGTTTCATC	59	GTGCATGAGTGGATTCAAGG	59	324
61	CCESSR111	122550	(TCA)6	CTTCATGGACCATTCTCACG	59	TAGCCGAAACAGAGCATTTG	59	290
62	CCESSR112	122619	(GAA)6	TCCCCTGAATTGGACTCTTC	59	ACAAAGGCCAACGTTTTACC	59	452
63	CCESSR113	122646	(AGC)6	GCATCCTTGTATTCGGAGGT	59	ACCACTCGCTTCAACCTCTT	59	115
64	CCESSR114	122681	(AAAG)5	CCAGGCTAAGTGCTCATCAA	59	GGCAGCGCAGTAAAGTCATA	59	209
65	CCESSR115	122758	(TC)13	CTCCCATTTCCTCTTTCTCT	55	TAGGGTTTTGAAGGGCAATC	59	274
66	CCESSR116	122793	(TC)18	GAGAAGTTTGCCAGAACCCT	58	ATCAATCTTCAAAGGGCCAC	59	453
67	CCESSR117	122797	(CT)12	CTCGTCCCTCTCCCTGTACT	58	ACCACAACAGCGAACATCAT	59	160
68	CCESSR118	122811	(AAG)7	CTTTCCTCAGCTTCACCACA	59	CCCCTGTTTCTGGTCTTGTT	59	371
69	CCESSR119	122842	(ATA)6	TGCAAGTGTGATGAATGTGG	59	CTAAGGCGAGAAAACAAGGG	59	431
70	CCESSR120	122880	(GAAGCA)4	TAGGGGGACCAATAGCAAAG	59	ACACGTCTTGCGACAAAGTC	59	212
71	CCESSR121	122996	(TTA)6	CCGGACAGGAACAAGAAAAT	59	CATTCAATTGCCTGACCATC	59	443
72	CCESSR122	123010	(TCC)6–30 bp-(TCA)7	GTCTGCATCTGCTTGCTCAT	59	AAACCATCTATCCCAGCCAG	59	438
73	CCESSR123	123023	(CTGCCT)4	GACAGAGGAATCCTTGCCAT	59	TCCCAGAAAAATCACCTTCC	59	494
74	CCESSR124	123110	(AACTA)4	GAGAAGCGAACCACACTTGA	59	ACTTGAGCAGCAACCCTTTT	59	183
75	CCESSR125	123132	(CTATGA)4	GAGCGCTATGGATGAGCATA	59	GCTGTACAGGATTGGGAGGT	59	478
76	CCESSR126	123134	(CTG)6	ACCACCACAAGCACAACAAT	59	TGACCCCCAACAAAACAAAG	61	457
77	CCESSR127	123160	(TA)11	CTGATGGGCGTCATCAATAC	59	GGGAAAGGGAGTCTCAACAA	59	394
78	CCESSR128	123185	(ACG)6	GAGGAAATTCGGGAAGTTGA	59	CATCGTCATCATCATCGTCA	59	280
79	CCESSR129	123240	(TTC)6	CCAGAACCAACTAGGGTTTT	56	TCACTTGAATTGCAGAAGGC	59	498
80	CCESSR130	123248	(GCA)6	TCTTCTACAGCTGCCACCAC	59	ATTGCGAAGAATGAAGGGTC	59	137
81	CCESSR131	123291	(CT)10	CTTCTCCAAACGGACCAAAC	60	CTCAGCCTCCATTGCACTAA	59	172
82	CCESSR132	123309	(AT)20	CGTAAATTAGGGGCGTTGTT	59	TTGGCACAAACCTGATGGTA	60	227
83	CCESSR133	123323	(ACAT)5	TCTCCCCATGGTACTTCACA	59	CCCATGCATTTCCAGACATA	59	463
84	CCESSR134	123329	(TC)13	TCACTGCCTGAGGCTTTATG	59	GAGCATGGTCCCGATAAGAT	59	380
85	CCESSR135	123332	(AG)10	TTGTTCTCTCCAGGGGAGTC	59	CGTAGAACCAATCCAGCAGA	59	210
86	CCESSR136	123342	(AC)9	AATATGAATGGAGGCGAAGG	59	ACAATGTCCCGAACTGTTGA	59	347
87	CCESSR137	123362	(AAT)6	GGTCTGAAAGTGGCCAAAAT	59	GGGAGATACGCTCAAGGTGT	59	496
88	CCESSR138	123381	(CGG)6	AAACCTAGTGGTGGAGGTGG	59	CAGCTTGGTTCCAGTCTCAA	59	240
89	CCESSR139	123445	(TC)9	AATCTTGTGGAGGAAATGCC	59	GCTTTTTGCTTCGGTACCTC	59	349
90	CCESSR140	123562	(CT)9	CGAGGCTCTATCCTCCTCTC	58	CTGACGCATCTGACCTCACT	59	182
91	CCESSR141	123682	(CTC)7–10 bp-(TTC)7	AGTACCTCGACAACCCCAAC	59	AGGCTCTGCCAAATCAAAGT	59	479
92	CCESSR142	123741	(CAGCAA)4	GAAGTTTGAAGCCTGAAGCC	59	TTCCGCGTATCTGAATCTTG	59	478
93	CCESSR143	123742	(TTGTA)4	AACCGATTTGATTCAGAGCC	59	CTCCACTCCACTGGGTTTTT	59	294
94	CCESSR144	123743	(TTGTA)4	AACCGATTTGATTCAGAGCC	59	AAAAAGGTCAAACGTCAGCC	59	269
95	CCESSR145	123795	(GCG)8	AGGCTTGAAGACGAAATGCT	59	GCTCTTTCCAGTCGATCTCC	59	309
96	CCESSR146	123812	(ATCTTT)4	ACACTCGACCCTCAAGTTCC	59	AGTGATCATCCATCCATCCA	59	482
97	CCESSR147	123819	(CTTC)5	ACGAGGGCAAAACAAAGAAG	59	TCAAGACGAAGAAATGCAGG	59	112
98	CCESSR148	123822	(GCT)6	ATCCGCGAAGCTACTTGTTT	59	ACGAATTTCTGGTCCACTCC	59	133
99	CCESSR149	123903	(CCG)6	GGCTCTGGAGGCTTTGAATA	59	GGCGAGCATGATTAGACAGA	59	256
100	CCESSR150	123909	(CT)14	CTTCCAGGTTCCTTGTCCTC	59	CCATCAGCACTTCCATCATC	59	389
101	CCESSR151	123967	(TTTC)5	CTCTTCCCTACTTTGCTGCC	59	AGGGTTTCTGTCGCTGTTCT	59	374
102	CCESSR152	123993	(TG)10	GGCCTAGTACTCCCAATCCA	59	ATGATGCAGTTGATCCTCCA	59	251
103	CCESSR153	124161	(CT)12	CTCGGCTCCCAAATATTCAT	59	ACATGCGGTTATGCACTCAT	59	453
104	CCESSR154	124171	(CCA)6	ACCACCACCTCCAGAGAATC	59	CGTAGAAATTGCTATCCGCA	59	421
105	CCESSR155	124195	(AGC)6	ATCCCCATCAGAAGACCTCA	59	GAAGCTCGACAAACAGGACA	59	296
106	CCESSR156	124198	(GAACT)4	CCCAGAAGAAATCCCAGTGT	59	GACCAATCGAGGCCAATAAC	59	240
107	CCESSR157	124268	(TGC)6	TTTCTCTCACACGCCAAAAG	59	GCTGATGATGCGATCAAAGT	59	406
108	CCESSR158	124269	(CCT)7	AGTCGTCTTCTGGTGGCTTT	59	GAGGAGGAGGTGGTGGTG	59	195
109	CCESSR159	124309	(AAAGG)4	TTCGAGTTCTATGGCTCGTG	59	TGGTGGTATGGAAAACAGGA	59	345
110	CCESSR160	124355	(CAAAA)4	TCGCCTTTTCTCTCACTTCA	59	GCTCGTTTCCTCTTGACTCC	59	359
111	CCESSR161	124358	(ACA)6	ATGAGGATGAGGAGGGATTG	59	TCCCTCAAGCTGTCTTTCCT	59	407
112	CCESSR162	124358	(AAG)6	AGCTTCCTCCAGAACCTCAA	59	ATCATCATCAGGGCCTCCT	59	147
113	CCESSR163	124365	(AAC)6	TTGCTCTTCCACTTTCATCG	59	CTTCACGAAATGCCTCAGAA	59	171
114	CCESSR164	124426	(TTTTTA)5	GACGTGGAGGGATCAGTTTT	59	CAATTCCGACGTTTCATCAG	59	140
115	CCESSR165	124428	(TTTTTA)4	GACGTGGAGGGATCAGTTTT	59	TGAATATGACCCGCATGAGT	59	239
116	CCESSR166	124436	(GAG)6	CGAGTACCATTGGGATGATG	59	ACTGTCATTTGAGTGCTCCG	59	304
117	CCESSR167	124457	(AC)9	CAAAGAAAAACCGCTCTCG	59	AGTGGAGGATTTGGAAAACG	59	367
118	CCESSR168	124461	(AATA)6	CTCTGCCCACTTTCTCCTTC	59	CACTTGGGCATCTGAGAAGA	59	354
119	CCESSR169	124465	(TTCA)6	TCCCCCATTCATTTGGTAGT	59	GCAAGGAGACCTTCAAGAGG	59	296
120	CCESSR170	124468	(TTCCTC)4–24 bp-(ATC)6	TCTCCCATTCATTCCAGGAT	59	GTAAGTGCGGATGATGATGG	59	342
121	CCESSR171	124565	(ATT)6	GCTCTGTTCAGGAAGGGAAG	59	GTATCAGAATCCCCACCCAA	60	406
122	CCESSR172	124577	(AAG)6	TGGCTTTTCTCCGTTATCCT	59	TTCTTCTTGGTGCTTGATCG	59	293
123	CCESSR173	124612	(CGG)6	TCCTTTCCTAGCCTTCTGGA	59	AGAGAAAAAGAGCGCGAGAG	59	331
124	CCESSR174	124697	(GAC)6	GAAAATCGAGAAAGGGTGGA	59	GAGTGGTCCTCAAGTCCCAT	59	242
125	CCESSR175	124700	(GCT)6	ACACCAACGACGGGTAAGAT	59	CAGCCAGATTCGAATACCCT	59	346
126	CCESSR176	124759	(AG)11	GGGATTGCTGCATAGGAAAT	59	CATCTCAATCAAATGCCCAC	59	290
127	CCESSR177	124765	(CCA)7a(AAC)6	CTGCGACTCAGACATCCACT	59	ACGCTGTCTCCTCCGTAACT	59	444
128	CCESSR178	124767	(TCAT)5	GCCGGCTAGTCTATACGGAG	59	ACCTTGAAGTTTGGTGGAGC	59	369
129	CCESSR179	124817	(TC)9	TCATTGCTGCTTCAGTCTGTC	59	TCTCTTCAAAGCGGATTCCT	59	354
130	CCESSR180	124851	(TTC)11	GACTTCTCTCTCCCCCTTCC	59	TTGGAGCGTAATGTCGTAGG	59	422
131	CCESSR181	124871	(AGC)6	ACCAATTTCAAACCTCCAGC	59	CGAACACCACATGCATTACA	59	475
132	CCESSR182	124949	(CCT)7	CCCCTTAAAATCGATTCCCT	59	CGGAGAGGAATTTTCGACAT	59	328
133	CCESSR183	124969	(AATTG)4	TCTAGCTTGAGGGGATCGTT	59	TAAGCCTAAGCAAAGCAGCA	59	243
134	CCESSR184	124995	(GAGAAG)4	CACCAAAGCCATGAAAGCTA	59	TCCCAAATCTCCTCTCCATC	59	224
135	CCESSR185	125025	(TC)11	CCTCCTTAAAACCCTAAACCG	59	TCTAGTATTTTGGTGGGGGC	59	302
136	CCESSR186	125054	(TA)9	TCTTGTCTTGCACATTTCCC	59	GGTCTCCGTTGTTGAGGATT	59	283
137	CCESSR187	125099	(ACC)6	TTCCCGCTTTACCAGATACC	59	TTCCTCCAACAGACAACAGC	59	388
138	CCESSR188	125102	(ACC)6	TTCCCGCTTTACCAGATACC	59	AAACATAAGGTTGGGGTGGA	59	281
139	CCESSR189	125104	(AT)11	CTGGCTCTGACATGCAATTT	59	TTCAGAAGCGCGTAGTCTGT	59	387
140	CCESSR190	125128	(TC)12	TGCCACTTTCTTCTCCTCCT	59	CTGGAAAAGGTGAAAAGGGA	59	418
141	CCESSR191	125139	(CAGCGG)4	AGCACATCCAACACCACAGT	59	AACCCAACGTTAAGGGACAC	59	204
142	CCESSR192	125151	(ACA)8	ATTCCGAATCGGGTACAGAG	59	AATGTACATCCCTTCCCCAC	59	400
143	CCESSR193	125186	(AAG)6	GCCTCAACCACTTGCCTATT	59	CGTGATCATGATGCCCTAAC	59	372
144	CCESSR194	125232	(AT)13	ATTCAGTTGCAGCTGTGGAG	59	ATGATCTGGGAAAGGACAGG	59	314
145	CCESSR195	125265	(GT)14	TGATGATAGCTCCGCTTTTG	59	AGCCAATCACCAGCAAGATT	60	254
146	CCESSR196	125286	(CTC)8	GGGTCCATGTTCGTCTTTTT	59	GAGACCAAGCATTGAAGCAA	59	304
147	CCESSR197	125289	(ATTTT)5	GAGGATCACTTTTGCCCCT	59	GAAAAAGGTCAAGACATCCC	56	470
148	CCESSR198	125382	(AC)16	CTCTCCCCATCCCTCAACT	59	GTGAAGGAGGTGGTGGTCTT	59	365
149	CCESSR199	125416	(AT)16	ATCATCCCCTCGATAGCAAC	59	TCCCGAAAGGAAAGAATGAG	59	183
150	CCESSR200	125444	(TG)11	TGGTTGTAGGTGGTCGTCAT	59	GCTCGGGGATATCGATCTTA	59	495
151	CCESSR201	125484	(CTCCGA)4	AGGGTTTTTCGGAGATGACA	60	CCGGGAAAACTAAGAGCAAG	59	424
152	CCESSR202	125529	(TC)9	GTGCTCGTGCAATTGAAACT	59	ATGCCGAGTGGATGTCTATG	59	490
153	CCESSR203	125676	(AAACAA)4	TGATCTTGATCCCCTCATCA	59	GATGCATTCACAAAACCGTC	59	393
154	CCESSR204	125734	(GAG)6	TCCTTGGCTCGAGATCTTTT	59	TTTGTGTCCTCTTCTCGCAC	59	434
155	CCESSR205	125776	(CT)14	AGGATTGCCTTCCGTTTGTA	60	GATTCAACTCGTGGGACCTT	59	225
156	CCESSR206	125893	(CAT)7	CCCCTTGTTTCTCTTTGTGG	60	TGCATCATCCCTTGTATCGT	59	271
157	CCESSR207	126139	(GGC)6	TCCGTTCCTCTTTCTCGTTT	59	CACAGCACCCATGGTAACAA	60	411
158	CCESSR208	126219	(GAG)6	TGGTCTTCAATCACCAAGGA	59	GACACATTGCACGTAGTCCC	59	134
159	CCESSR209	126243	(CAC)8	GGTGGACGAGGCTTTTATGT	59	CTGTGAGCAAACTTGACGGT	59	180
160	CCESSR210	126250	(CAC)9	GCCAAAATCCCTGTTCTCAT	59	TGTGGATGCACCAGATTCTT	59	245
161	CCESSR211	126255	(GAA)6	ACGAAAGGGTTGAAGGTGAC	59	GACAGAGACGACGAAGACCA	59	412
162	CCESSR212	126427	(ATC)7	TCCAATGGTTTACAGGAGCA	59	TCTCCGGTGTAAAACTGCTG	59	341
163	CCESSR213	126427	(CAGGAG)6	GGCGGCTTCGGTATTATTTA	59	GCACTAGTTTCTTGGACTGGG	59	184
164	CCESSR214	126506	(TTTC)6	TTCATTGTCTTTGAGCCAGG	59	CCCCATCACTCATTCCTTCT	59	339
165	CCESSR215	126540	(AAG)9	AAATCGAATGGCTTGGAGAC	59	GTGCAAGTAAATCCGAGCAA	59	309
166	CCESSR216	126661	(AGT)6	TTTGTCATTTCTCTCGCTGC	59	AGTTGGGGAGAATTGACCTG	59	440
167	CCESSR217	126701	(TTCA)5	AAAGGATACGGCTCACAAGG	59	GAATGAATGAACGAGGGGTT	59	124
168	CCESSR218	126701	(TTCA)6	AAGGAGCCAATCGTTCATTC	59	TTACCCTGTAGGTTGGTGGAG	59	175
169	CCESSR219	126730	(CT)10	GAGTTGGCTTGAAAGCATGA	59	CCATCCCCAAGGAAAGTGTA	60	371
170	CCESSR220	126767	(GA)9	GCACAAGACCTTCCCTTTGT	59	CTCTTGCACGTCGTTTTGTT	59	428
171	CCESSR221	126881	(GCA)6	CGGTGCTGCAGTTTCACTAC	60	CAAGGCAGCAAGTATACCGA	59	351
172	CCESSR222	126910	(AAT)8	CCAACAGAAAAGTTGGGGAC	59	TTAGCCGAATCGTTATTCCC	59	163
173	CCESSR223	127055	(TTC)6	GAAATCCCACTCGGTGAAGT	59	CCCACCCCGAAGATATAAAA	59	401
174	CCESSR224	127171	(AAGG)5	ATTTCAGTCTGGGTCTTCCC	58	TGAGGCAGACCTTGTTCTTG	59	470
175	CCESSR225	127376	(GT)10	TCCCAGAACAAAACGAGACA	59	CTTGTCGTAAACGGCTTCAA	59	270
176	CCESSR226	127478	(CT)23	CAGCCTCCGGTTAGCAAG	60	TGTCCAATACCCACTCTTCG	59	179
177	CCESSR227	127479	(ACC)10	GTACCCCCACAACACTCACA	59	GATGCTTCTCATCGTCTCCA	59	404
178	CCESSR228	127712	(TTTA)5	GATTTTGTGCCAAAGGGAAG	60	GCTTTGACCGGAGAAGTCAT	59	115
179	CCESSR229	127774	(CCT)6	CCGCCGTTAAATCAAACTCT	59	CAAAGAAAGCTGCTCACTGC	59	120
180	CCESSR230	127785	(AT)13	GCTGCTGCTTTGCTTGTTCT	61	AGCTTCAGAAATGGTGGCAT	60	333
181	CCESSR231	127800	(GAA)9	GCTACAGCGTGCAGAAAAAG	59	TACTCCTCAGGCCTCCATTT	59	318
182	CCESSR232	127828	(TCA)6	ACCTGGGAGGACTGATAACG	59	CCAGATTCCATACTTGGGCT	59	177
183	CCESSR233	127910	(AAAAGG)4	GAAGACTCTGTAAAGGAACGACC	58	GTGTTATGGCTCAACCATGC	59	347
184	CCESSR234	127938	(CTG)8	GCGGCTTAGTTCTATCCTCG	59	ATGGCTGACAGCTTCCTCTT	59	442
185	CCESSR235	127992	(TAA)6	AGTGGGAAAAGTGAAGGTGG	59	ATTAATCCCCTCCTCCGAGT	59	225
186	CCESSR236	128005	(CTC)8	AGTCAGGTATGCTGCCATTG	59	GGAAATCTTTGGGGTCTGAA	59	322
187	CCESSR237	128130	(CCT)6	CGTCAATAAATTGGTGTGGC	59	GGGAGAATGGGAGATGAAGA	59	142
188	CCESSR238	128268	(TA)11	CCGTCGTTACTGCAAAAGAA	59	ACAGAGGGGTTTCAAACAGG	59	254
189	CCESSR239	128301	(AAGAAC)4	TCCCAAGTCCCTATCTCTGC	59	CAGCTGATGGTTTAGGAGCA	59	123
190	CCESSR240	128306	(TCT)7	TCTGTGTCCCTTTTCAGCAG	59	GGATGGTCAATGCAGATGAG	59	221
191	CCESSR241	128309	(CGG)8	ATATCATGGATGGTGCTGCC	61	TCCAGATTGTGCTGCTTTTC	59	355
192	CCESSR242	128315	(TTC)6	GCACGAGGCTTTCTCTCTTT	59	CAGCAGGAAACTGGAGAAGTC	59	260
193	CCESSR243	128377	(AG)12	GGTTCTTCTGTGTGGGTGTG	59	GTAGAAGCCAGCTAATGCCC	59	107
194	CCESSR244	128432	(TG)15	CACACACCCAACACACAGAA	59	GCGAGAGGAATTTTGACCAG	60	482
195	CCESSR245	128504	(GAG)6	TCCATCCCATATTCCCATCT	59	GTCGGGTCTCTTCTTCTTGC	59	454
196	CCESSR246	128527	(GA)10	CACCAACAATTCCCTATCCC	59	TCCATCTCCAACACTACCCA	59	427
197	CCESSR247	128527	(CTG)6	CACCAACAATTCCCTATCCC	59	TCCATCTCCAACACTACCCA	59	427
198	CCESSR248	128527	(CTG)6	TGGGTAGTGTTGGAGATGGA	59	CAATGACCGCCTGTATTCTG	59	260
199	CCESSR249	128550	(ATC)6	CTAGTAGCCATGGTCACCCA	59	TTCTTCGTAGGCAGCTCTGA	59	416
200	CCESSR250	128587	(TC)11	GAGGCAAACCAAATGGAGAT	59	ATTGAAAAGCCTGCTGAACC	59	358
201	CCESSR251	128673	(CT)9	TTCTCTGCTGCTGCTCATTT	59	GGCACACATTTATCCACTCG	59	245
202	CCESSR252	128894	(AAAAG)4	GCCCAAAGCTGTTAGAGACC	59	TCCTTGTCATACGCTTGCTC	59	344
203	CCESSR253	129060	(TCT)7	CAATTTGCAGTCAGTCCGTT	59	ACATCTGCCACTGTCTGAGC	59	216
204	CCESSR254	129129	(GAG)7	ACAGATGCTTTATGGGAGGG	59	GGAAAGGTATTGCTGGGGTA	59	272
205	CCESSR255	129269	(GCA)9	TGAAGGTTCACTGATTGGGA	59	TGCTGTTGTAACTGCTGCTG	59	477
206	CCESSR256	129431	(AAG)9	CGCCACCAGTACAGATCAAG	59	TCCGCATATCGAACACTCAT	59	496
207	CCESSR257	129439	(CA)10	AGTCACCACACAGATGCTGC	60	GGCCATTCAAGGTTTCTTGT	59	296
208	CCESSR258	129524	(GAA)8	CTAATCTTCCACGGCTCTCC	59	TTTTGTTCAGCAGCAAGGTC	59	412
209	CCESSR259	129581	(AAAAG)4	CCAACCTCACGTCTCACCTA	59	GCCAGTGACACAGACGACTT	59	102
210	CCESSR260	129646	(TC)11	CTTCAGCCACAACAGATGCT	59	AAAAGAGCACACAGTGCAGC	59	179
211	CCESSR261	129668	(CT)12	CATGATTATGCGTTGGCTTC	59	GGTTGTCATGTTGAATGAGG	56	163
212	CCESSR262	129745	(CAC)10	GCCTCCAGTCAACAAACAAA	59	TGGTGCTTCTCTTCCTTGTG	59	252
213	CCESSR263	129793	(CA)10	TAGCGGGGAAAATTGATAGG	59	AGGGGCTTTTTCTCCATCTT	59	487
214	CCESSR264	129797	(TCC)6	TCGATGATGGCTACAGCTTC	59	TTGCTCCTGAGAAACACTGG	59	312
215	CCESSR265	129906	(TA)11	GACCAGAGAGAGACACCTACTTTT	57	CTCTTCCTAGCAGCAGCAGA	59	186
216	CCESSR266	129913	(CAG)6	TTCCTTTAAAACCGTCAGGG	59	CTGCATGCTCTGTTGTTCCT	59	264
217	CCESSR267	129917	(AAGAGC)5	GAGATGGAGAGACCATCATCC	58	AACTAAGACTTTTGCCGCGT	59	267
218	CCESSR268	129950	(TCCACT)4	TTCCCCATCTAAGGCAAAGT	59	CTGATTCAGGGCTCTCCATT	59	403
219	CCESSR269	129957	(GAGCAG)4	AACCCACCTTCCTCTCCTTT	59	CATGCATATCGGACACAACA	59	294
220	CCESSR270	129964	(GAT)7	AAGGCTCGACATGTTTTTCC	59	CCTACGCAACATCATCAACC	59	221
221	CCESSR271	129972	(AG)9	CTTTCCGCCTTTTCGTACTC	59	AACACACGCACAACACACAG	59	392
222	CCESSR272	130030	(AG)19	GGCTGCAAGAACATAGAGCA	59	GAAATCCTTGTTGGCTGGTT	59	369
223	CCESSR273	130066	(GAG)6	AAAGAAAGAGCCCTCCACAA	59	GCTCCAAAACCACCATTACC	59	212
224	CCESSR274	130235	(TGC)6	GGTTTATGACGTTGTGGACG	59	GGCCTATGATGCGGTAGAAT	59	198
225	CCESSR275	130276	(GGC)7	GGCGGCTACTCAGAGACTTC	59	TGGAACTCCAATTCCTTTCC	59	300
226	CCESSR276	130329	(AT)13	AGGCTGCGTTCCATATAAGA	57	CCCAGAAACTCCATTTCGAT	59	410
227	CCESSR277	130342	(ATC)6	CTCTGCAGAACAACCCTTCA	59	CCAACAGCTAAAAGTGCCAA	59	296
228	CCESSR278	130353	(CA)11	TCTCTCTCTCTCCATTCGCA	59	GGTACGAGGTTGATCGGAGT	59	154
229	CCESSR279	130353	(CAC)6	ATGGCTCCATCATCTCATCA	59	ATTGGTGGGTCCTTCAATTC	59	215
230	CCESSR280	130368	(AG)17	GGCTGTCTTAAGGCCCTAGTT	59	ATCAAGTGATGCTGTCGAGC	59	436
231	CCESSR281	130475	(CTC)7	CGCCAACACCCCTTATCTAT	59	CATTGAGGAATTGGAACGTG	59	485
232	CCESSR282	130516	(CCT)6	ACCCTGCTCCACCTTATCAC	59	GACAGAAACCTTCATCCCGT	59	357
233	CCESSR283	130575	(AGA)7	GTGGTCAGTATGGTTGCTGG	59	AGCCACAAAAGAACCATTCC	59	456
234	CCESSR284	130635	(AG)13	AGCACCGTCAGACTCTCCAT	60	TAAGCCAAACGTCGTCGTAG	59	283
235	CCESSR285	130647	(CAC)6	GAGGCCGAGTTACGAGAGTC	59	AGCATGCCAACCCTTTATTC	59	366
236	CCESSR286	130785	(TAAAA)4	TGCTCGGACCCTCTAAGTTT	59	TGGAGTCGTTTAAAAAGGGG	59	334
237	CCESSR287	130804	(AG)10	CGTTACAGAATTTGCGGATG	59	CAACTCTAAGCCGTCGATGA	59	360
238	CCESSR288	130947	(GCAGAG)4	TTTTGAACACGTCAAGGCTC	59	CGAAGAGGGGACTGAGAAAG	59	273
239	CCESSR289	130972	(TCA)9	GCACAACCCATATGACGAAG	59	GGAGGAACAGTTGGAGAATGA	59	107
240	CCESSR290	130978	(AG)16	TTATTATTGCACAGCCCCG	60	AACAATCCATTCTTGCTCCC	59	388
241	CCESSR291	130993	(TC)14	CGGTGGGGTTCTGATAATTT	59	CGGTTTCAAGCTAACGAACA	59	313
242	CCESSR292	131127	(TC)11	ATCGTGTTTAGCAGTGACGC	59	GCAGATATCATATACTCCCGTC	55	143
243	CCESSR293	131130	(AAG)6	CACTCACTCAACACTTCGCA	59	CCATCTTCCACCACCTTTCT	59	426
244	CCESSR294	131306	(TCGCA)4	CTTTGGGGGCTGAGAGTAAG	59	GCTCCATATTGCAACGTCTT	58	432
245	CCESSR295	131334	(CCA)7a(AAC)6	TAATACATCCACTCCGCCAA	59	TACTCATCAACAACCTCGCC	59	320
246	CCESSR296	131479	(TG)9	GCAATTGTGGATGGTCTGAG	59	AAACACACTGGGAAAGAGCA	58	424
247	CCESSR297	131560	(ACC)6	CAATCTGTTCCCACGTATGC	59	ACAAGTGCTCCAACAAACCA	59	328
248	CCESSR298	131583	(AT)12	CCACACACTGCAGATTGGTT	60	AAGATCTCTGGGGCATCAAC	59	453
249	CCESSR299	131633	(CTT)6	AGGTTCGAGCCAGGAAGATA	59	TTGCCTTGGTTGATGACTTC	59	141
250	CCESSR300	131669	(AG)12	TCCGGGAAGGATAGTGGTAG	59	TTGCTGAAACAGTCTCCCTC	58	203
251	CCESSR301	131714	(GA)22	ATTAGTGCGGAATTCCCTTG	59	TGATAATCTCGAGTGACCGC	59	491
252	CCESSR302	131742	(TA)18	TAATGCACGCACAAACACAC	59	ATCATTGCAAATCTCCTCCC	59	112
253	CCESSR303	131761	(CA)10	CATGGATTGGAATTCTGCTG	59	CAGATTAGCTCCGCAATCAA	59	454
254	CCESSR304	131806	(ATG)8	GCACCTAAGAGAGGAGTGCC	59	ATGAGTTTGGCTGGATGTGA	59	401
255	CCESSR305	131831	(GCCTGC)4	CCGATTTCGCTCGTAGTGTA	59	TATCAATACGGACTGCTGGG	59	462
256	CCESSR306	131899	(GAT)9	TCCGCTGGTCACAAGAAAT	59	ATCAAACTTCGACACCCCTC	59	184
257	CCESSR307	131901	(AAC)6	AAACTTTGATATCCCGGCTG	59	GCCGCCTTTTGTAGAAGAAC	59	175
258	CCESSR308	131958	(TCA)9	GATTCCAGTATTTCGGCTCC	59	TTGCTCAGATTAGGCTGTCG	59	211
259	CCESSR309	131966	(AC)10	TTTGAACTTGCTGGAGCATC	59	TGGTGAACTTCTGTTTTCCC	58	392
260	CCESSR310	132036	(AAG)6	CTCCTCAACACTCCTGACCA	59	CCCTCCTGAAGCTGCTTATC	59	194
261	CCESSR311	132205	(GT)10	CCTTGATTGTCACGTGTATGC	59	AAGATATGCCCAGGTCATGC	60	424
262	CCESSR312	132207	(TCGT)7	AAAGGATACGGCTCACAAGG	59	ACCCTGTAGGTTGGTGGAGA	59	198
263	CCESSR313	132325	(AAG)8	TGAGCACTCAAGGGAAATTG	59	TTCGTCCAGGGAGTAGCTTT	59	462
264	CCESSR314	132326	(AAG)6	GCTTTTGTCCAAGGGATGAT	59	GGAGCTCCCAGGAGGTAGTA	58	336
265	CCESSR315	132398	(TA)11	ACGTTTATTACGGCGGATTC	59	ATCGCCGTCCCATAACTATT	58	174
266	CCESSR316	132466	(AGA)6	TCTGTCTGGGCAACACTATAC	55	AGAAAGGGAGGTGGAGGAAT	59	338
267	CCESSR317	132484	(CT)9	TTTGCAGTTATGACTTGGGC	59	TCCTCACTGGAAAGGGATTC	59	449
268	CCESSR318	132502	(GTCTT)4	GGGGATCAGCGTAAGAATGT	59	TTTCCACCCACGTAATAGCA	59	346
269	CCESSR319	132516	(TCA)6	CCGTCCACGTTCACATCTAC	59	AGTAGCGCGTAGTGACCTGA	59	130
270	CCESSR320	132606	(AGC)6	GACCAGAAGAACAGCGATGA	59	CTCCTTCTTCTTCTCGGCAG	59	285

*: Unigene ID as per downloaded from the SGN ftp site ftp://ftp.sgn.cornell.edu/coffee/.

### Types, Frequency and Distribution of SSRs in the coffee transcriptome

The coffee EST unigene database analyzed here, comprised 13,175 unigenes having a total length of 8923 kb and an average lengthof ca. 677 bases/unigene [15]. These ESTs were found to contain a total of 2,589 SSRs (having a minimum numbers of repeats as: six for DNRs, four for TNRs and three for all other HO-NRs) located in 2,028 unigenes. The identified 2,589 SSRs comprised- 502 DNRs, 1285 TNRs, 503 TtNRs, 144 PNRs and 155 HNRs, which differed significantly in their relative distribution and abundance accross the unigene ESTs (Tables S1 & S2). The mean length of repeat iterations (RI) for all the SSRs was 5.2, whereas average length of DNRs was maximum (9.6 RI) followed by TNRs (4.6 RI). Among the individual SSRs, AC had the maximum average repeat length of 10.5 RI, followed by AG (8.6 RI), AT (8.3 RI), CG (6.3 RI); all the TNRs had RI in the range of 4.3 to 4.8, whereas all other larger SSRs had an average RI of less the three. The identified SSRs having a repeat core of 18 bp or more were selected as candidate ‘usable’ SSRs for further primer designing/marker conversion. Overall, *in-silico* analysis of the EST unigenes revealed one EST-SSR (having a minimum repeat core of 12 bp) per 3.4 kb and one usable SSR (having a minimum repeat core of 18 bp) per 15.9 kb of robusta transcriptome (Table S2). Among the individual SSRs, the most abundant EST-SSR motif was AG, followed by AAG.

### Development of microsatellite markers from usable EST-SSRs

Only 483 (18.7%) of the total 2589 identified SSRs had a repeat core>18 bp, which were used for marker conversion. Primer pairs could be designed for 320 of these SSRs, of which randomly chosen 50 pairs were further tested for validation studies. These included SSRs with DNRs (30%), TNRs (64%), PNRs, HNRs (2% each) and complex SSRs (see Table 1 for marker ID, primer sequences, repeat motifs, amplicon size, sequence ID and functional identity). Of the selected 50 primer pairs, 44 could be successfully amplified as single locus SSR marker, indicating 88% primer to marker conversion ratio. Considering this high conversion ratio, another ca. 200 useful genetic markers are expected from the remaining 270 putative EST-SSRs (Primer IDs: CCESSR51 to CCESSR320) that are lsited in Table 3.

### Identification and development of genomic SSRs using affinity capture

Sequencing of randomly chosen 66 recombinant clones from the small-insert SSR-enriched robusta genomic library (prepared in this study), revealed 81 potential SSRs distributed in 62 sequences. A redundancy analysis of these sequences indicated presence of a total of 60 non-redundant sequences of which 56 were SSR +ive (93.3% of non-redundant sequences) containing 72 non-redundant SSRs. Non-redundant dataset contained 10 sequences with more than one SSR either in compound formation or separated by>50 bp distance. The non-redundant SSRs contained 56.9% AG, 33.3% AC, 2.8% AT, 2.8% AAC, 1.4% AAG and 2.7% A/T repeat motifs (Table S3).

From the 56 SSR+ive sequences, a total of 41 primer pairs could be designed successfully (with five pairs containing two SSRs each). Of these, 25 pairs (encompassing 28 SSRs) resulted in robust PCR amplifications (Table 2), and all of them could further be validated as single locus markers indicating ∼61% primer to marker conversion ratio.

### Validation of EST- SSRs for use in genetic studies

**Figure 1:**
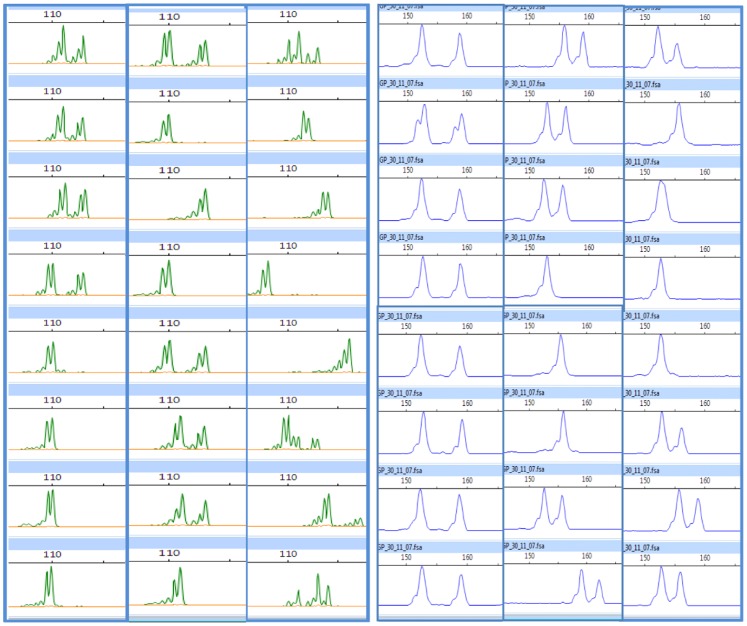
Representated Gene Scan profiles showing the SSR alleles obtained using the new SSR markers for some of the coffee genotypes tested in the study for marker validation. The right side set of 3 panels is for the genomic-SSR CCRM-33, and the similar set on the left is for genic-SSR CCESSR05. The 3 panels in each set represent 8 genotypes each of arabica and robusta coffee, and 8 of the related coffee species, respectively, from right to left end.

All the new 44 EST-SSRs resulted in good amplicons exhibiting low to medium allelic diversity when tested on a panel of 16 elite robusta and arabica genotypes (Figure 1). Overall, a maximum of six and seven alleles (N_A_) with an average of 2.1 and 3 alleles/SSR were obtained for the tested markers of which 65.9% and 81.8% were polymorphic/informative for tetraploids and robusta genotypes, respectively (Table 4). Fifteen markers in the case of tetraploids and eight for robustas were found to be monomorphic. Moreover, 14 markers resulted in double alleles (i.e. consistent presence of two allelic amplicons across the tested samples) indicative of duplicated loci in case of all the tested tetraploid arabicas. In general, no private alleles were evident except in one robusta genotype (Sln274) for marker CCESSR14.

**Table 4:** Allelic diversity attributes of the newly developed 44 EST-SSRs when tested over cultivated and wild related coffee genera.

Species:	*C. arabica* (n = 8)						*C. canephora* (n = 8)						*Coffea* spp. (n = 12)			*Psilanthus* spp. (n = 2)		
Primer ID	N_A_	PA	Allele range (in bp)	*H_o_*	*H_e_*	PIC	N_A_	PA	Allele range (in bp)	*H_o_*	*H_e_*	PIC	N_A_	PA	Allele range (in bp)	N_A_	PA	Allele range (in bp)
CCESSR01	2	0	251–254	0.00	0.50**	0.36	2	0	251–254	0.50	0.40	0.30	7	3	246–260	4	0	246–281
CCESSR02	5	0	181–209	0.33	0.67*	0.57	5	0	183–207	0.38	0.71*	0.62	10	0	178–209	1	0	183
CCESSR03	6	0	236–260	DL			5	0	232–244	1.00	0.78	0.68	12	1	232–260	3	0	226–242
CCESSR04	2	0	252–255	0.00	0.50**	0.36	2	0	252–255	0.50	0.40	0.30	6	2	247–261	2	0	247–252
CCESSR05	2	0	153–159	DL			4	0	153–162	0.63	0.68	0.61	4	0	153–162	2	0	154–159
CCESSR06	1	0	223	MM			6	0	225–242	0.88	0.72	0.63	17	5	105–243	2	0	225–227
CCESSR07	2	0	211–133	0.38	0.33	0.26	2	0	211–233	0.50	0.40	0.30	8	4	211–239	4	4	220–240
CCESSR08	2	0	151–167	DL			2	0	164–167	0.50	0.40	0.30	7	1	151–176	2	0	161–167
CCESSR09	3	0	138–202	0.00	0.62**	0.50	5	0	129–212	0.50	0.76*	0.67	10	0	129–212	1	0	188
CCESSR10	3	0	158–235	0.83	0.68**	0.55	2	0	221–138	1.00	0.53*	0.38	13	3	158–247	3	0	212–227
CCESSR11	3	0	147–155	DL			3	0	147–155	0.38	0.54	0.43	4	0	147–160	1	0	147
CCESSR12	1	0	218	MM			1	0	218	MM			4	1	205–221	1	0	208
CCESSR13	4	0	122–140	0.75	0.59	0.51	4	0	131–141	0.50	0.73	0.62	11	3	115–144	3	0	122–130
CCESSR14	5	0	243–257	0.50	0.61	0.54	6	1	231–256	0.43	0.60	0.54	14	0	145–257	2	0	231–136
CCESSR16	1	0	190	MM			1	0	190	MM			1	0	190	1	0	181
CCESSR17	2	0	182–191	0.88	0.53	0.37	3	0	179–191	0.50	0.58	0.48	5	2	179–191	2	0	182–185
CCESSR18	1	0	169	MM			1	0	169	MM			1	0	169	2	1	169–172
CCESSR19	3	0	230–236	DL			4	0	224–234	0.75	0.70	0.56	9	1	224–239	4	0	224–232
CCESSR20	2	0	153–162	DL			4	0	153–166	0.88	0.74	0.64	8	2	153–189	2	1	166–177
CCESSR21	2	0	170–175	0.00	0.23	0.19	2	0	170–175	0.00	0.40*	0.30	9	4	168–211	4	1	168–176
CCESSR22	1	0	158	MM			7	0	159–180	1.00	0.85	0.77	12	2	158–180	2	0	151–153
CCESSR23	2	0	168–174	0.88	0.53	0.37	2	0	174–177	0.00	0.23	0.19	7	1	166–178	3	1	167–172
CCESSR26	4	0	214–227	0.86	0.78	0.67	3	0	214–227	0.57	0.47	0.39	9	3	212–236	1	0	218
CCESSR27	1	0	132	MM			5	0	122–139	0.50	0.65	0.56	13	5	115–195	3	0	122–195
CCESSR28	1	0	197	MM			1	0	189	MM			5	1	186–213	2	2	194–207
CCESSR29	3	0	96–106	DL			5	0	96–111	1.00	0.75*	0.66	5	0	96–111	3	0	96–106
CCESSR31	1	0	150	MM			4	0	150–182	0.63	0.74	0.64	7	2	140–182	2	2	167–182
CCESSR32	1	0	181	MM			4	0	179–185	0.38	0.66*	0.57	12	5	177–204	2	1	187–189
CCESSR33	2	0	236–250	DL			2	0	236–241	0.88	0.53	0.37	6	0	230–256	2	0	223–236
CCESSR34	2	0	185–191	0.13	0.53*	0.37	2	0	174–185	0.13	0.13	0.11	10	3	135–224	2	2	193–222
CCESSR35	1	0	115	MM			4	0	122–128	0.75	0.73	0.62	9	1	115–130	2	0	111–120
CCESSR36	1	0	112	MM			2	0	112–114	0.25	0.23	0.19	4	1	102–124	2	0	102–114
CCESSR38	2	0	177–179	0.63	0.46	0.34	3	0	177–181	0.25	0.24	0.21	11	4	170–185	2	0	177–179
CCESSR39	1	0	150	MM			1	0	147	MM			2	0	147–150	2	0	143–150
CCESSR40	2	0	220–222	DL			3	0	220–223	0.25	0.24	0.21	7	3	217–115	1	0	222
CCESSR41	3	0	212–219	DL			4	0	214–223	0.71	0.76	0.65	9	0	212–226	1	0	139
CCESSR42	1	0	129	MM			1	0	129	MM			5	1	120–141	1	0	132
CCESSR43	2	0	184–190	DL			3	0	187–199	0.33	0.32	0.27	5	0	178–199	3	0	174–187
CCESSR44	3	0	250–259	0.88	0.58	0.45	3	0	238–259	1.00	0.59*	0.46	8	1	236–259	3	0	247–159
CCESSR45	2	0	144–151	DL			1	0	151	MM			8	3	137–166	2	0	125–151
CCESSR47	1	0	211	MM			1	0	211	MM			2	0	211–213	1	0	207
CCESSR48	2	0	199–211	DL			2	0	192–205	0.13	0.13	0.11	4	0	192–211	1	0	182
CCESSR49	2	0	192–203	DL			3	0	192–203	0.50	0.43	0.35	7	1	188–203	2	0	185–197
CCESSR50	1	0	122	MM			2	0	116–122	0.38	0.33	0.26	4	0	116–128	2	0	122–128
**Range**	***1–6***	***0***		***0.00–0.88***	***0.23–0.78***	***0.19–0.67***	***1–7***	***0–1***		***0.00–1.00***	***0.13–0.85***	***0.11–0.77***	***1–17***	***0–5***		***1–4***	***0–4***	
**Average**	***2.14***	***0.00***		***0.47***	***0.54***	***0.43***	***3.00***	***0.02***		***0.54***	***0.53***	***0.44***	***7.52***	***1.57***		***2.11***	***0.34***	
**SD (±)**	***1.21***	***0.00***		***0.36***	***0.13***	***0.16***	***1.57***	***0.15***		***0.26***	***0.22***	***0.19***	***3.62***	***1.59***		***0.89***	***0.81***	
**SE (±)**	***0.18***	***0.00***		***0.10***	***0.03***	***0.03***	***0.24***	***0.02***		***0.04***	***0.04***	***0.03***	***0.55***	***0.24***		***0.14***	***0.12***	

**Note**: N_A_: Number of amplified alleles; PA: Number of Private Alleles; H_o_: Observed heterozygosity; H_e_: Expected heterozygosity; PIC: Polymorphism Information Content; PI: Probability of Identity; NA: Not amplified; *: Significant HW dis-equilibrium at P<0.05; **: Highly significant HW dis-equilibrium at P<0.01; The putative DL (duplicated loci) markers were not considered for calculation of various estimates as these appear to be fixed exhibiting no segregation.

The PIC values were comparable (0.19–0.67 and 0.11–0.77), and no significant differences were seen in the observed/expected heterozygosity (*H_o_/H_e_*: t-value = 0.70; P = 0.49; and t-value = 0.68; P = 0.40) for the new markers across the tested tetraploids and diploid robustas, respectively. However, significant differences were observed in the total number of amplified alleles (N_A_: t = 3.74, P<0.005), as well as, the behaviour of the polymorphic markers (P*m*s) when tested for HWE and LD in the tested tetraploids and the robusta genotypes (Table 4). In general, more markers were in HWE and only a relatively small proportion of markers exhibited LD and heterozygote excess and/or deficiency in case of robustas, in comparision to tetraploid arabicas.

### Validation of genomic SSRs for use in genetic studies

A total of 25 putative genomic SSRs were also validated as genetic markers (Table 5). When tested on the panel of 16 elite robusta and arabica (tetraploid) genotypes, five of these markers in arabicas and one in robustas were found to be monomorphic. Twelve ofthe polymorphic markers in arabicas resulted in double alleles (putative duplicated loci). In total, a maximum of seven and eight alleles (N_A_) with an average of 2.7 and 4.3 alleles/marker were obtained for the tested polymorphic markers of which 32% and 96% were informative in arabicas and robustas, respectively (Figure 1). The PIC values varied considerably, with mean PIC value being 0.47 (range 0.12–0.78) for tetraploids, which was significantly less than 0.60 (0.12–0.85) observed for robusta (Table 5). Further, the Student’s t-test revealed significant differences in N_A_ (t = 4.09, P = 0.00) but non-significant differences in PIC estimates (t = 1.26, P = 0.13), as well as, for the observed/expected heterozygosity estimates *(H_o_*/*H_e_*) for the comparable markers of arabica and robusta genotypes.

**Table 5:** Allelic diversity attributes of the newly developed 25 genomic SSRs when tested over cultivated and wild related coffee genera.

Species:	*C. arabica* (n = 8)						*C. canephora* (n = 8)						*Coffea* spp. (n = 12)			*Psilanthus* spp. (n = 2)		
Primer ID	N_A_	PA	Allele range (in bp)	*H_o_*	*H_e_*	PIC	N_A_	PA	Allele range (in bp)	*H_o_*	*H_e_*	PIC	N_A_	PA	Allele range (in bp)	N_A_	PA	Allele range (in bp)
CCRM02	3	0	252 −262	DL			3	0	256– 268	0.71	0.69	0.67	7	2	252– 278	2	1	162 −272
CCRM06	2	1	143 −156	0.14	0.14	0.12	1	0	143	MM			1	0	143	1	0	143
CCRM07	5	0	124– 140	0.71	0.82	0.78	5	1	124– 146	1.00	0.78	0.74	6	3	115– 136	2	0	124 −128
CCRM10	3	0	96– 106	0.29	0.38	0.32	2	0	105– 106	0.00	0.23	0.23	6	2	98– 100	NA	–	–
CCRM14	3	0	109– 188	DL			7	2	110–132	0.63	0.88**	0.83	8	5	109–155	1	0	123
CCRM15	1	0	298	MM			3	0	243– 249	0.17	0.62**	0.61	7	2	243– 361	NA	–	–
CCRM16	3	0	198– 202	0.00	0.70**	0.67	8	0	190– 240	0.88	0.89	0.84	11	3	180– 226	3	0	180 −192
CCRM17	1	0	230	MM			7	2	226– 248	1.00	0.86	0.81	10	4	216– 250	2	1	210 −238
CCRM19	4	0	217– 234	0.43	0.69	0.62	7	1	196– 252	0.83	0.92	0.85	13	4	188– 246	3	0	210 −226
CCRM21	7	1	256– 290	DL			7	0	256– 288	0.63	0.90	0.85	16	6	234– 320	2	0	258 −320
CCRM22	3	0	194– 203	DL			5	1	194– 254	0.25	0.67**	0.63	10	4	194– 254	2	1	201 −226
CCRM23	2	0	140– 162	DL			3	0	140– 151	0.38	0.49	0.46	7	1	140– 162	2	0	140– 162
CCRM24	3	0	202– 213	DL			3	0	211– 215	0.63	0.57	0.56	8	1	202– 217	2	0	205– 213
CCRM28	2	0	203– 206	0.00	0.26	0.23	2	0	202– 206	0.13	0.13	0.12	6	0	202– 210	3	0	202– 210
CCRM31	1	0	111	MM			4	0	101– 110	0.38	0.44	0.42	7	1	101– 115	2	2	113– 118
CCRM33	2	0	110– 116	0.14	0.14	0.33	3	0	110– 116	0.88	0.64	0.64	8	1	106– 122	2	0	112– 116
CCRM34	2	0	130–146	DL			4	0	144– 163	0.75	0.75	0.72	9	1	130– 146	3	1	146– 163
CCRM35	1	0	156	MM			3	2	152– 156	0.00	0.43**	0.42	1	0	156	1	0	156
CCRM36	2	0	139–146	DL			7	1	139– 175	1.00	0.90	0.85	11	3	139– 183	1	0	139
CCRM37	2	0	140– 150	DL			3	0	140– 152	1.00	0.63	0.63	4	1	140– 150	2	1	140– 142
CCRM38	4	1	175–217	DL			3	1	175– 206	0.38	0.34	0.33	12	5	163– 214	2	0	175– 200
CCRM40	2	0	144–151	DL			5	0	159– 177	0.63	0.72	0.68	8	0	151– 177	2	0	151– 153
CCRM41	1	0	101	MM			2	0	95– 101	0.14	0.14	0.14	2	0	95–110	1	0	101
CCRM42	5	0	130– 161	DL			5	1	138– 157	0.75	0.75	0.72	13	2	121– 165	4	0	134– 155
CCRM45	3	0	183– 195	0.86	0.69	0.64	5	0	187– 212	0.50	0.76	0.72	7	1	183– 201	2	0	187– 189
**Range**	***1 −7***	***0 −1***	***–***	***0 −0.71***	***0.14 −0.81***	***0.12 −0.78***	***1 −8***	***0 −2***	***–***	***0.00 −1.00***	***0.13 −0.92***	***0.12 −0.85***	***1 −16***	***0 −6***	***–***	***0 −4***	***0 −2***	***–***
**Mean**	***2.68***	***0.12***	***–***	***0.32***	***0.45***	***0.47***	***4.28***	***0.48***	***–***	***0.57***	***0.63***	***0.60***	***7.92***	***2.08***	***–***	***2.04***	***0.30***	***–***
**SD (**±**)**	***1.46***	***0.33***	***–***	***0.32***	***0.28***	***0.24***	***1.97***	***0.71***	***–***	***0.33***	***0.25***	***0.22***	***3.67***	***1.78***	***–***	***0.77***	***0.56***	***–***
**SE (**±**)**	***0.30***	***0.07***	***–***	***0.07***	***0.06***	***0.05***	***0.40***	***0.15***	***–***	***0.07***	***0.05***	***0.05***	***0.75***	***0.36***	***–***	***0.16***	***0.11***	***–***

**Note**: N_A_: Number of amplified alleles; PA: Number of Private Alleles; H_o_: Observed heterozygosity; H_e_: Expected heterozygosity; PIC: Polymorphism Information Content; PI: Probability of Identity; NA: Not amplified;

**: Highly significant HW dis-equilibrium at P<0.01; The putative DL (duplicated loci) markers were not considered for calculation of various estimates as these appear to be fixed exhibiting no segregation.

Further, it was notable that while>83% of the P*m*s were in HWE and only few markers showedsignificant heterozygote deficiency to varying extent in both arabicas and robustas, the number of marker-pairs that exhibited LD was significantly more in arabicas (28.0%; 8 of 28 pairs) that that seen in robustas (14.2%; 36 of 254 marker pairs).

### Mapping of new EST- and genomic SSRs

**Figure 2:**
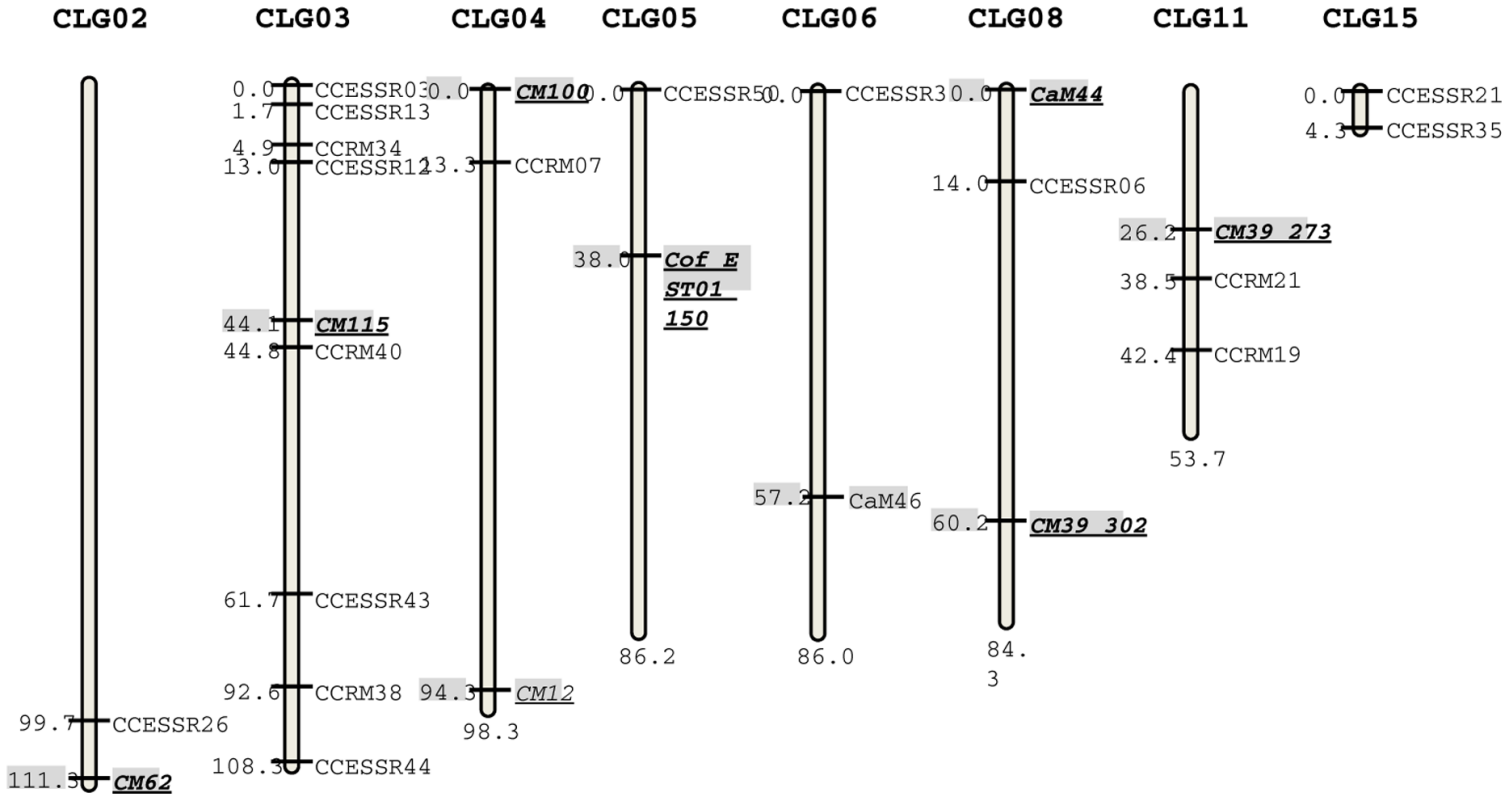
Map positions of 18 new SSR markers developed in this study (11 CCESSR and 7 CCRM markers) on robusta linkage map; mapping population was derived from a cross between CxR (a commercial robusta hybrid) and a local selection Kagganahalla [26]. The SSR markers of the existing map used as anchor markers are shown in italic and bold face.

The 69 new SSR markers were also tested for their suitability in linkage mapping. In total, 11 of the 44 EST-SSRs (∼39%) and seven of the 25 genomic SSRs (28%) could be mapped onto an existing first-generation framework linkage map of robusta coffee [2], [26]. This map comprised a total of 374 mapped markers (71 SSRs, 185 RAPDs and 118 AFLPs) on 11 major and 5 minor linkage groups. The new markers developed in the present study were mapped using the existing SSRs on the map as anchor markers. The 18 new markers that could be mapped, occupied positions on eight distinct linkage groups, with eight markers on CLG03; two markers each on CLG06, CLG11, CLG15; one marker on CLG02, CLG04, CLG05, CLG08 (Tables 1 & 2). The position of these 18 markers on robusta linkage groups alongwith positions of SSRs used as anchores (CM62, CM115, CM12, CM100, Cof_EST01_150, CaM46, CaM44, CM39_302, CM39_273) is shown in figure 2.

### Cross-species/−genera transferability and marker conservation

New SSR-markers when tested on 13 related *Coffea* and two *Psilanthus* species, exhibited robust cross-species amplifications with alleles of comparable sizes in the tested taxa (Figure 1, Tables 4 & 5, Table S4). The EST-SSRs showed 100% transferability accross the tested *Coffea* and *Psilanthus* spp., whereas the genomic-SSRs indicated 96% amplification and transferability for *Coffea* spp. and 92% for the related *Psilanthus* spp. The analysis also indicated some private alleles (PAs), which possibly could be species-specific (Tables 4 & 5).

### Generic affinities within/between cultivated and wild coffee germplasm by new SSRs

The SSR allelic data were examined for their utility in ascertaining the genetic diversity and generic inter-relationships between the cultivated, as well as, the wild coffee genepool. The average genetic distance values calculated using the EST-SSR allelic data were in general, significantly less but comparable to that obtained using the genomic SSRs for the tested arabicas, robustas, as well as, for different *Coffea* and *Psilanthus* species.

**Figure 3:**
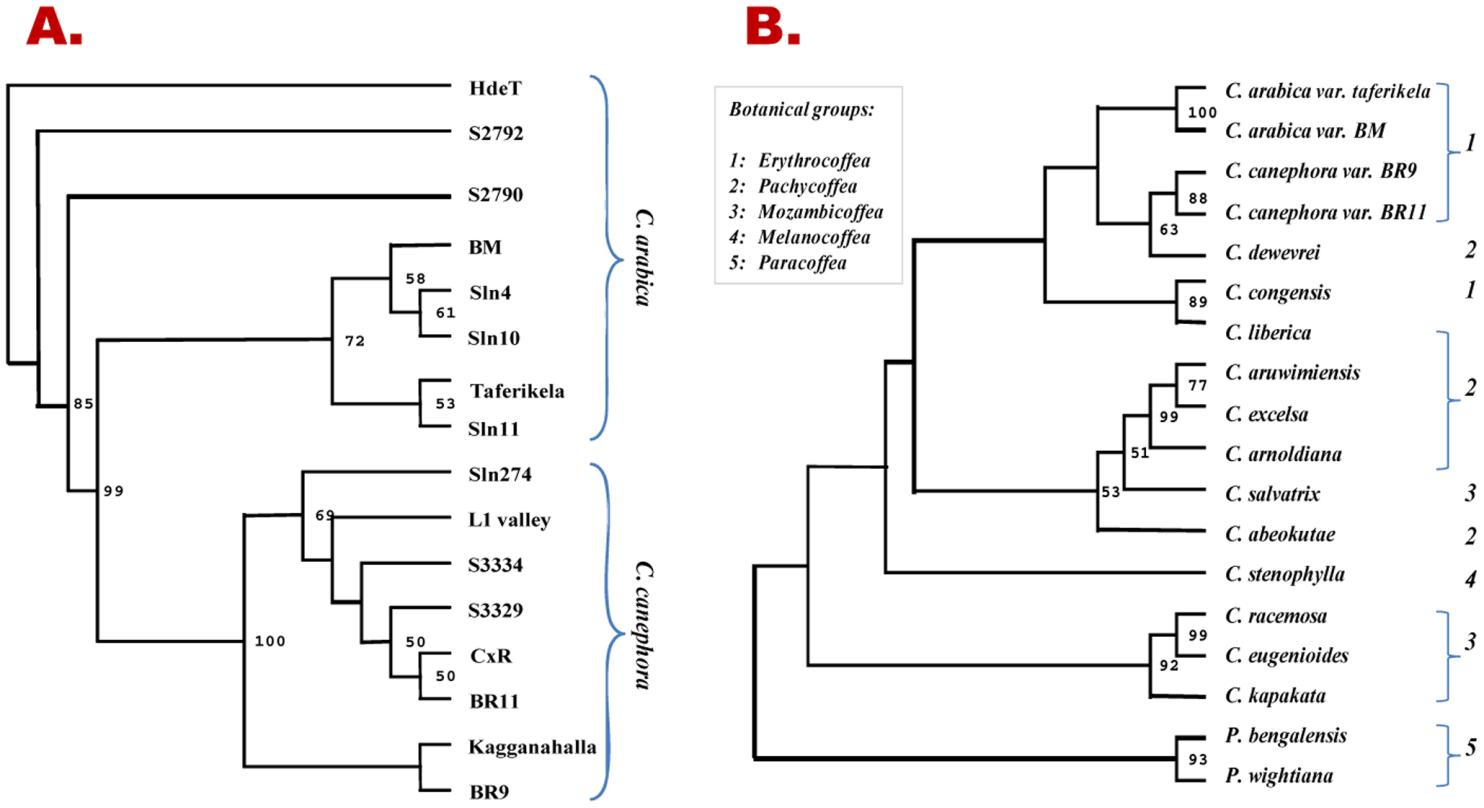
Unrooted phenetic trees based on the allelic diversity across the tested 44 EST-SSRs showing generic affinities between the: a) *C. arabica* and *C. canephora* genotypes, and b) 14 *Coffea* and two *Psilanthus* taxa; (only>50 bootstrap values are shown).

The NJ phenetic tree generated using the genetic distance estimates of EST-SSRs allelic data clearly resolved the tested germplasm in two distinct clusters, one representing all the tetraploid arabica*s,* while the other comprised all the diploid robusta genotypes (Figure 3a) with significant branch support. The selections formed a single cluster within the tetraploids cluster, while pure arabicas and hybrid-selections appeared in distict sub-clusters. Similarly, in clustering analysis of 14 related species (12 *Coffea* and two *Psilanthus* spp.; Figure 3b) along with two genotypes each from *C. arabica* and *C. canephora,* tetraploid Erythrocoffeas (*C. arabica*) and diploid Erythrocoffea (*C. canephora*) formed coherent clusters. Moreover, the grouping of the related taxa, in general, was as per their botanical type with few changes. Though all the entries from Erythrocoffeas fell into one cluster, it contained two entries from Pachycoffeas (*C. dewevrei* with *C. canephora* and *C. liberica* with *C. congensis*). The remaining four of the Pachycoffeas (*C. excelsa*, *C. arnoldiana*, *C. aruwemiensis*, *C. abeokutae*) grouped with each other with good bootstrap support. The *C. salvatrix* a Mozambicoffea was also grouped with these Pachycoffeas, while the other three Mozambicoffeas (*C. racemosa*, *C. eugenioides*, *C. kapakata*) and two Paracoffeas (*Psilanthus* spp.) appeared as independent strong groups. Single Melanocoffea species (tested in this study), *C. stenophylla* was not grouped with any of the above species cluster but was found close to the *Coffea* species than the *Psilanthus* spp.

Similar results were obtained using the data from genomic SSRs (CCRMs, data not shown).

## Discussion

### SSR motifs in coffee transcriptome, and development of new EST-SSR markers

In the present study, 15.4% of the coffee ESTs were found to contain SSRs, which is comparable with our earlier study [11], but much higher than 2.7–10.8% that was reported for 18 representative dicotyledonous species [27], and 7 −10% reported for monocot species [28]. Notwithstanding this apparent enrichment/higher abundance, the SSRs in coffee transcriptome were very comparable to other plant species in observations like: ***1.*** Abundance of TNRs than DNRs; ***2.*** Abundance of AG among the DNRs followed by AT; ***3.*** CG as the least abundant among the DNRs; ***4.*** Abundance of AAG among TNRs (among the dicots); ***5.*** Predominance of GC-rich TNRs (but not CCG/GGC) than the non-GC-rich TNRs.

A total of 18.7% of the detected EST-SSRs were found to be suitable candidates for primer design, a comparatively lower proportion (∼50%) then we reported earlier [11]. The main attributes that rendered majority of the identified SSRs unsuitable for marker development were: a shorter repeat core (<18 bp) and/or flanking sequences of low complexity (AT/GC-rich and/or regions prone to secondary structure formation) or shorter lengths seriously constraining designing of optimal primer pairs. However, in this study, primer-to-marker conversion ratio (ca. 88%), was higher than many earlier similar studies in other crops. Such differences in marker conversion ratios are expected due to differences- in the quality of primers designed), GC content of the genome, the genome complexity, and/or genome size [13].

### SSR enrichment and development of genomic SSRs

The genomic DNA library constructed in this study, resulted in very high proportion of SSR+ive sequences, with very low degree of redundancy (9.1%; 6 out of 66 identified SSRs positive sequences). This was notable, as in earlier similar studies the apparent high success rates were generally confounded by high degree of redundancy [29]. Similarly, the proportion of SSR positive sequences found suitable for primer designing was also higher (87%) in our study than the average of 54±3% recorded in other species [30]. These obsrevationssuggest that the enrichment approach used in this study may be a desirable strategy for efficiently entrapping and targeting the SSRs even in genome(s) like coffee that are relatively poor in SSR motifs [2].

### Utility of new EST- and genomic SSRs as genetic markers

The SSRs provide desirable markers for studying genetic diversity, germplasm characterization, constructing reference panels/bar codes, for individualization of genotypes, linkage mapping, population biology, and taxonomic relationships of related taxa [2]. Therefore, it becomes desirable to validate the new markers for their utility in genetic studies, which unfortunately has been lacking in majority of published studies describing development of coffee-specific SSR markers.

Various genetic parameters *viz*., allelic diversity, PIC, *H_o_*, *H_e_*, HWE, LD calculated for all the new EST and genomic SSRs and mapability on linkage map, amply suggested their possible utility as genetic markers (see Table 4 & 5). In general, the extent and pattern of allelic/genetic diversity revealed by the new markers conform to that reported earlier for the coffee genomic SSRs [5], [6], [31], and the EST-SSRs [8], [11].

Different genetic parameters/tests such as *H_o_*, *H_e_*, LD, HWE are important indicators of origin, evolution and distribution of diversity in the available genepool. The heterozygosity measures (*H_o_, H_e_*) for the new SSR markers indicated heterozygote decay (deficiency) in the tested germplasm. The HWE and LD analysis of the polymorphic markers were in general agreement with our earlier observations with genomic as well as EST-SSRs [5], [8], [11]. Overall, these studies indicated that the tested robusta germplasm comprised allogamous, relatively unrelated genotypes, while autogamous tetraploids comprised mostly of hybrid varieties/selections with overlapping/shared pedigrees. The results thus suggest the suitability of the new markers for reliably ascertaining genetic diversity in the coffee gene pool.

### Cross-species/−generic transferability

All the new EST- and genomic SSR markers revealed very high and robust cross species/−generic amplifications with alleles of comparable sizes when tested on 12 other *Coffea* and two *Psilanthus* taxa. The data revealed that the markers described here show much higher taxa transferability than earlier published genomic−/EST-SSR markers [2], [5], [7], [11]. This is significant as successful cross-species amplification is generally restricted to related species within a genus and reduces when tested for different genera [32]. Further, it was interesting to note that the new SSRs that were monomorphic/uninformative for the tested arabica/robusta germplasm, exhibited considerable polymorphism across the tested related taxa (the only exceptions were the marker CCESSR16 and 18 that showed a very low conservation even across the *Coffea* spp.). Thus the new SSR markers described here strengthen the possibility of their use as Conserved Orthologous Sets (COS) for genetic characterization of different related wild coffee taxa, and also for coffee taxonomic/synteny studies.

### Diversity analysis and genetic relatedness within/between *Coffea* and *Psilanthus* species

The EST−/genomic-SSRs described in this study were able to group all the 16 genotypes (representing the cultivated genepool) in phenetic clustering that was indicative of their species status and known pedigrees (Figure 1a). Similarly, the analysis 14 *Coffea* and two *Psilanthus* species, revealed generic affinities that were largely in agreement with their known taxonomic relationships (Figure 1b), based on their geographical distribution as well as Chevalier’s botanical classification [33]. Importantly, the analysis distinctly separated the two Paracoffea species (*P. bengalensis* and *P. wightiana*) from all the other *Coffea* spp. These results are similar to the earlier published studies undertaken to ascertain species relationships using SSRs [2], [7], [8], [11], as well as other marker approaches [34]–[36]. These results, thus, amply demonstrate that the new SSR markers developed in the present study can be considerably informative in exploring the taxonomic relationship of coffee species complex.

## Conclusions

The present study describes a total of 69 new validated SSRs; 44 EST-SSRs developed from coffee transcriptome using *in-silico* methodology, and 25 genomic SSRs developed using SSR enrichment approach. In addition, it provides primer pairs for additional 270 putative EST-SSRs. Analysis of the identified SSR-positive ESTs also provided insights into the relative abundance and distribution pattern of different SSR motifs in the coffee transcriptome, which was found to be relatively rich in its SSR abundance. Among the identified EST-SSRs, TNRs followed by DNRs were more abundant than other SSRs, and among different types of SSR motifs, AG was the most abundant. All the 69 markers were found to be polymorphic in the tested coffee/related germplasm and their utility as efficient genetic markers could be demonstrated for diversity analysis, germplasm individualization, linkage mapping, cross-species transferability and taxonomic studies. As many of these SSRs showed a very high cross-species transferability, they can aid in conservation, management and resolving taxonomic relationships, as Conserved Orthologous Sets (COS) for *Coffea* and *Psilanthus* species and more importantly as efficient, and informative genetic landmarks on molecular linkage maps.

## Supporting Information

Table S1 **Summary statistics of screening of the coffee unigene ESTs for SSRs.**
(PDF)

Table S2 **Summary statistics of distribution and abundance of detected SSRs in the unigene ESTs and SSR frequency estimates for coffee transcriptome.**
(PDF)

Table S3 **Characteristics and distribution of the detected SSR motifs (without MNRs) across the non-redundant 56 SSR+ive sequences generated using SSR enrichment approach.**
(PDF)

Table S4 **Inter-species and inter-generic transferability of the new EST-SSRs and genomic SSR markers.**
(PDF)
