# Supplementary material for: Development of Genic and Genomic SSR Markers of Robusta Coffee (Coffea canephora Pierre Ex A. Froehner)
Source: PLoS One. 2014 Dec 2;9(12):e113661. doi: 10.1371/journal.pone.0113661 (PMC4252042; doi:10.1371/journal.pone.0113661)
Supplement: Table S1 — Summary statistics of screening of the coffee unigene ESTs for SSRs. (PDF) [file pone.0113661.s001.pdf]

Development of genic and genomic SSR markers of robusta coffee (*Coffea canephora* Pierre ex A. Froehner)

Prasad S. Hendre, Ramesh K. Aggarwal
Centre for Cellular and Molecular Biology (CSIR-CCMB), Uppal Road, Hyderabad- 500 007, Telangana, India

---

Table S1: Summary statistics of screening of the coffee unigene ESTs for SSRs

Total number of unigene ESTs analysed (X)	13,175
Total length of unigene ESTs analyzed (Kb)	8,922.8
Average size of unigene EST (bases)	677.3
Total number of SSRs identified (EST-SSRs; Y)	2,589
Total number of unigene ESTs containing SSRs (SSR-ESTs)	2,028 (15.4% of X)
Number of unigenes containing > 1 SSR core	405 (3.1% of X)
Number of unigenes containing compound SSRs	217 (1.6% of X)
Total number of ‘usable’ SSRs[u]* (A)	483 (18.7% of Y)
Total number of unigenes containing usable SSRs	457 (3.5% of X)
Number of unigenes containing > 1 usable SSR core	24 (0.2% of X)
Number of unigenes containing usable compound SSRs	10 (<0.1% of X)
Number of SSRs found suitable for primer designing (B)	318 (12.3% of Y; 65.8% of A)
Number of primer pairs used for validation studies	50 (15.7% of B)
Number of working primer pairs of the tested pairs	44 (13.8% of B)
Number and average spacing (in Kb) between identified SSRs[+]	2589, 3.4
DNRs	*502, 17.7*
TNRs	*1285, 6.9*
TtNRs	*503, 17.7*
Number and average spacing (in Kb) between usable SSRs[+]	483, 15.9
DNRs	*201, 32.2*
TNRs	*195, 45.6*
TtNRs	*26, 342.3*

*: *SSRs having a repeat core of minimum 18 bp; +: The calculations include DNRs, TNRs, TtNRS, as well as PNRs, and other HNRs.*
