# Supplementary material for: Development of Genic and Genomic SSR Markers of Robusta Coffee (Coffea canephora Pierre Ex A. Froehner)
Source: PLoS One. 2014 Dec 2;9(12):e113661. doi: 10.1371/journal.pone.0113661 (PMC4252042; doi:10.1371/journal.pone.0113661)
Supplement: Table S2 — Summary statistics of distribution and abundance of detected SSRs in the unigene ESTs and SSR frequency estimates for coffee transcriptome. (PDF) [file pone.0113661.s002.pdf]

Prasad S. Hendre, Ramesh K. Aggarwal
Centre for Cellular and Molecular Biology (CSIR-CCMB), Uppal Road, Hyderabad- 500 007, Telangana, India

Table S2: Summary statistics of distribution and abundance of detected SSRs in the unigene ESTs and SSR frequency estimates for coffee transcriptome

SSR motif	No.s of SSRs detected (% of total SSRs)			Mean no. of repeats/SSR^d (Range of repeat iterations in the SSR core)	Estimated number and spacing of definable SSRs in the analyzed coffee transcriptome (a= 8.9 Mb)		Estimated number and spacing of usable SSRs in the analyzed coffee transcriptome (a= 8.9 Mb)	
	Definable SSRs (SSR^d)	Usable SSRs (SSR^u)	Proportion of usable versus definable SSRs (SSR^u/SSR^d)		No. of SSRs^d/Mb transcriptome (X^d = SSR^d/a)	SSR^d spacing in transcriptome (Kb) (Z^d = 1000/X^d)	No. of SSRs^u/Mb transcriptome (X^u = SSR^u/a)	SSR^u spacing in transcriptome (Kb) (Z^u = 1000/X^u)
DNRs								
AG	295 (11.4%)	140 (29.0%)	0.47	8.6 (6 to 21)	33.1	30.2	19.3	51.7
AT	123 (4.8%)	36 (7.5%)	0.29	8.3 (6 to 23)	13.8	72.4	7.2	139.1
AC	80 (3.1%)	25 (5.2%)	0.31	10.5 (6 to 36)	9.0	111.3	4.5	222.5
CG	4 (0.2%)	--	--	6.3 (6 to 7)	0.4	2225.0	--	--
**All DNRs**	**502 (14.6%)**	**201 (41.6%)**	**0.40**	**9.6 (6 to 36)**	**56.4**	**17.7**	**31.0**	**32.2**
TNRs								
AAG	282 (10.9%)	55 (11.4%)	0.20	4.8 (4 to 11)	31.7	31.6	6.2	161.8
ACC	183(7.1%)	16 (3.3%)	0.09	4.5 (4 to 10)	20.6	48.6	1.8	556.3
AGG	174 (6.7%)	31 (6.4%)	0.18	4.6 (4 to 8)	19.6	51.1	3.5	287.1
ACT	136 (5.3%)	6 (1.2%)	0.04	4.3 (4 to 9)	15.3	65.4	0.7	1483.3
AGC	120 (4.6%)	23 (4.8%)	0.19	4.8 (4 to 9)	13.5	74.2	2.6	387.0
ACG	104 (4.0%)	14 (2.9%)	0.13	4.5 (4 to 8)	11.7	85.6	1.6	635.7
AGT	102 (3.9%)	17 (3.5%)	0.17	4.6 (4 to 9)	11.5	87.3	1.9	523.5
AAT	87 (3.4%)	14 (2.9%)	0.16	4.6 (4 to 12)	9.8	102.3	1.6	635.7
CCG	54 (2.1%)	9 (1.9%)	0.17	4.6 (4 to 8)	6.1	164.8	1.0	988.9
AAC	43 (1.7%)	10 (2.1%)	0.23	4.6 (4 to 8)	4.8	207.0	1.1	890.0
**All TNRs**	**1285 (49.6%)**	**195 (40.4%)**	**0.15**	**4.6 (4 to 12)**	**144.4**	**6.9**	**21.9**	**45.6**
TtNRs								
AAAG	103	4 (0.8%)	0.04	3.2 (3 to 6)	11.6	86.4	0.4	2225.0

	(4.0%)							
AAAT	77 (3.0%)	4 (0.8%)	0.05	3.3 (3 to 6)	8.7	115.6	0.4	2225.0
AAAC	35 (1.4%)	1 (0.2%)	0.03	3.1 (3 to 5)	3.9	254.3	0.1	8900.0
AAGG	31 (1.2%)	3 (0.6%)	0.10	3.4 (3 to 8)	3.5	287.1	0.3	2966.7
AAGT	23 (0.9%)	5 (1.0%)	0.22	3.7 (3 to 6)	2.6	387.0	0.6	1780.0
AACT	22 (0.8%)	1 (0.2%)	0.05	3.2 (3 to 5)	2.5	404.5	0.1	8900.0
ACAT	20 (0.8%)	2 (0.4%)	0.10	3.3 (3 to 5)	2.2	445.0	0.2	4450.0
AGGG	19 (0.7%)	2 (0.4%)	0.11	3.3 (3 to 5)	2.1	468.4	0.2	4450.0
AATC	15 (0.6%)	--	--	3.2 (3 to 4)	1.7	593.3	--	--
AAGC	14 (0.5%)	2 (0.4%)	0.14	3.4 (3 to 7)	1.6	635.7	0.2	4450.0
AGGT	14 (0.5%)	1 (0.2%)	0.07	3.1 (3 to 5)	1.6	635.7	0.1	8900.0
AACC	13 (0.5%)	--	--	3	1.5	684.6	--	--
AATT	13 (0.5%)	--	--	3	1.5	684.6	--	--
AGAT	12 (0.5%)	--	--	3.1 (3 to 4)	1.3	741.7	--	--
ACGT	10 (0.5%)	1 (0.2%)	0.10	3.1 (3 to 5)	1.1	890.0	0.1	8900.0
AACG	10 (0.4%)	--	--	3.1 (3 to 4)	1.1	890.0	--	--
AATG	10 (0.4%)	--	--	3.3 (3 to 4)	1.1	890.0	--	--
AGCT	10 (0.4%)	--	--	3.2 (3 to 4)	1.1	890.0	--	--
ACCT	8 (0.3%)	--	--	3	0.9	1112.5	--	--
ACTC	8 (0.3%)	--	--	3.1 (3 to 4)	0.9	1112.5	--	--
ACAG	7 (0.3%)	--	--	3.1 (3 to 4)	0.8	1271.4	--	--
ACCC	6 (0.2%)	--	--	3.2 (3 to 4)	0.7	1483.3	--	--
AGGC	5 (0.2%)	--	--	3.2 (3 to 4)	0.6	1780.0	--	--
ACCG	4 (0.2%)	--	--	3	0.4	2225.0	--	--
ACGC	3 (0.1%)	--	--	3	0.3	2966.7	--	--
AGCC	3 (0.1%)	--	--	3.7 (3 to 4)	0.3	2966.7	--	--
CCCG	3 (0.1%)	--	--	3	0.3	2966.7	--	--
ACTG	2 (0.1%)	--	--	3	0.2	4450.0	--	--
AGCG	2 (0.1%)	--	--	3	0.2	4450.0	--	--
AGTC	1 (<0.1%)	--	--	3	0.1	8900.0	--	--
**All TtNRs**	**503 (19.4%)**	**26 (5.4%)**	**0.05**	**3.2 (3 to 8)**	**56.5**	**17.7**	**2.9**	**342.3**
**All PNRs**	**144 (5.6%)**	**26 (5.4%)**	**0.18**	**3.2 (3 to 5)**	**16.2**	**61.8**	**2.9**	**342.3**
**All HNRs**	**155 (6.0%)**	**35 (7.2%)**	**0.23**	**3.3 (3 to 6)**	**17.4**	**57.4**	**3.9**	**254.3**
Total SSRs	***2589***	***483***	***0.19***	***5.2 (3 to 36)***	***290.9***	***3.4***	***62.7***	***15.9***

Note: SSR^d: definable SSRs; SSR^u: usable SSRs; --: Not detected
