# Supplementary material for: Development of Genic and Genomic SSR Markers of Robusta Coffee (Coffea canephora Pierre Ex A. Froehner)
Source: PLoS One. 2014 Dec 2;9(12):e113661. doi: 10.1371/journal.pone.0113661 (PMC4252042; doi:10.1371/journal.pone.0113661)
Supplement: Table S3 — Characteristics and distribution of the detected SSR motifs (without MNRs) across the non-redundant 56 SSR+ive sequences generated using SSR enrichment approach. (PDF) [file pone.0113661.s003.pdf]

Development of genic and genomic SSR markers of robusta coffee (*Coffea canephora* Pierre ex A. Froehner)

Prasad S. Hendre, Ramesh K. Aggarwal
Centre for Cellular and Molecular Biology (CSIR-CCMB), Uppal Road, Hyderabad- 500 007, Telangana, India

Table S3: Characteristics and distribution of the detected SSR motifs (without MNRs) across the non-redundant 56 SSR+ive sequences generated using SSR enrichment approach.

SSR motif type/ length	**AG^T**	**AC^T**	**AT^{NT}**	***DNRs***	**AAC^T**	**AAG^T**	***TNRs^T***	***All detected SSRs (without MNRs)***
6 to 7-mer	5	4	1	*10*	1	1	*2*	***12***
8 to 10-mer	11	8	1	*20*	1	--	*1*	***21***
11 to 13-mer	6	5	--	*11*	--	--	--	***11***
14 to 16-mer	3	3	--	*6*	--	--	--	***6***
17 to 19-mer	2	1	--	*3*	--	--	--	***3***
20 to 22-mer	3	2	--	*5*	--	--	--	***5***
23 to 25-mer	7	0	--	*7*	--	--	--	***7***
26 to 28-mer	3	1	--	*4*	--	--	--	***4***
29 to 31-mer	1	0	--	*1*	--	--	--	***1***
Total number	41	24	2	*67*	2	1	*3*	***70***
Total length (RIs)	632	298	14	*944*	16	6	*22*	***966***
Mean length (RIs)	15.4	12.4	7	*14.2*	8	6	*7.3*	***13.8***

Note: ^{T}: Targeted SSR motifs; ^{NT}: Non-targeted SSR motifs; --: Not detected; RI: Repeat Iterations
