# Supplementary material for: Development of Genic and Genomic SSR Markers of Robusta Coffee (Coffea canephora Pierre Ex A. Froehner)
Source: PLoS One. 2014 Dec 2;9(12):e113661. doi: 10.1371/journal.pone.0113661 (PMC4252042; doi:10.1371/journal.pone.0113661)
Supplement: Table S4 — Inter-species and inter-generic transferability of the new EST-SSRs and genomic SSR markers. (PDF) [file pone.0113661.s004.pdf]

Development of genic and genomic SSR markers of robusta coffee (*Coffea canephora* Pierre ex A. Froehner)

Prasad S. Hendre, Ramesh K. Aggarwal, Centre for Cellular and Molecular Biology (CSIR-CCMB), Uppal Road, Hyderabad- 500 007, Telangana, India

---

Table S4: Inter-species and inter-generic transferability of the new EST-SSRs and genomic SSR markers

Species	*Coffea spp.*																	*Psilanthus* spp.			**Average Average C_{taxa} for all coffees**
	Erythrocoffea			Mozamicoffea					Melanocoffea	Pachycoffea								Paracoffea			
Primer name	*C. araica*	*C. congensis*	**Average C_{taxa}**	*C. eugenioides*	*C. kapakata*	*C. racemosa*	*C. salavatrix*	**Average C_{taxa}**	*C. stenophylla*	*C. excelsa*	*C. lierica*	*C. aeokutae*	*C. dewevrei*	*C. arnoldiana*	*C. aruwemiensis*	**Average C_{taxa}**	**Average C_{taxa} (*Coffea*)**	*P. engalensis*	*P. wightiana*	**Average C_{taxa} (*Psilanthus*)**	
a. EST-SSRs (*in-silico* EST database)																					
**All the CCESSRs**	1.00	1.00	1.00	1.00	1.00	1.00	1.00	1.00	1.00	1.00	1.00	1.00	1.00	1.00	1.00	1.00	1.00	1.00	1.00	1.00	1.00(**$C_{taxa\text{-}mark}$**)/1.00 (**$C_{mark\text{-}taxa}$**)
b. genomic-SSRs (SSR enrichment approach)																					
CCRM06, CCRM14, CCRM16, CCRM17, CCRM19, CCRM21, CCRM23, CCRM24, CCRM28, CCRM31, CCRM33 to CCRM38, CCRM40, CCRM42, CCRM45	+	+	1.00	+	+	+	+	1.00	+	+	+	+	+	+	+	1.00	1.00	+	+	1.00	1.00
CCRM02	+	+	1.00	+	+	+	+	1.00	+	+	+	+	+	-	+	0.83	0.85	+	+	1.00	0.87
CCRM07	+	-	0.50	+	+	+	+	1.00	+	+	+	+	+	-	+	0.83	0.77	+	+	1.00	0.80
CCRM10	+	+	1.00	+	+	+	+	1.00	+	+	+	+	+	+	+	1.00	0.92	-	-	0.00	0.80
CCRM15	+	+	1.00	-	+	-	+	0.50	-	-	+	+	+	+	-	0.67	0.69	-	-	0.00	0.60
CCRM22	+	+	1.00	+	+	+	-	0.75	+	+	+	+	+	+	-	0.83	0.77	+	+	1.00	0.80
CCRM41	+	+	1.00	+	-	+	+	0.75	-	+	+	-	+	+	+	0.83	0.85	+	+	1.00	0.87
**Average T_{mark}**	1.00	0.96	0.98	0.96	0.96	0.96	0.96	0.96	0.92	0.96	1.00	0.96	1.00	0.92	0.92	0.96	0.96	0.92	0.92	0.92	0.95(**$C_{taxa\text{-}mark}$**)/0.94 (**$C_{mark\text{-}taxa}$**)

+/-: Indicates 'amplification'/'No amplification' and are given a weightage of 1 and 0 for transferability/conservation calculations respectively; Tmark: Marker transferability over all the taxa; Ctaxa: Marker conservation over all the taxa; Tmark-taxa: Marker transferability of all the markers over all the taxa; Ctaxa-mark: Primer conservation across all the taxa over all the markers.
